# Cell-intrinsic insulin signaling defects in human iPS cell–derived hepatocytes in type 2 diabetes

**DOI:** 10.1172/JCI183513

**Published:** 2025-04-15

**Authors:** Arijeet K. Gattu, Maria Tanzer, Tomer M. Yaron-Barir, Jared L. Johnson, Ashok Kumar Jayavelu, Hui Pan, Jonathan M. Dreyfuss, Lewis C. Cantley, Matthias Mann, C. Ronald Kahn

**Affiliations:** 1 Section of Integrative Physiology and Metabolism, Joslin Diabetes Center, and; 2 Metabolism Unit and Division of Endocrinology, Massachusetts General Hospital, Harvard Medical School, Boston, Massachusetts, USA.; 3 Department of Proteomics and Signal Transduction, Max Plank Institute of Biochemistry, Martinsried, Germany.; 4 Advanced Technology and Biology Division, Walter and Eliza Hall Institute of Medical Research, Melbourne, Victoria, Australia.; 5 Department of Medical Biology, University of Melbourne, Melbourne, Victoria, Australia.; 6 Columbia University Vagelos College of Physicians and Surgeons, New York, New York, USA.; 7 Department of Cell Biology,; 8 Dana-Farber Cancer Institute, and; 9 Bioinformatics and Biostatistics Core, Joslin Diabetes Center, Harvard Medical School, Boston, Massachusetts, USA.

**Keywords:** Endocrinology, Hepatology, Metabolism, Adult stem cells, Diabetes

## Abstract

Hepatic insulin resistance is central to type 2 diabetes (T2D) and metabolic syndrome, but defining the molecular basis of this defect in humans is challenging because of limited tissue access. Utilizing inducible pluripotent stem cells differentiated into hepatocytes from control individuals and patients with T2D and liquid chromatography with tandem mass spectrometry–based (LC-MS/MS–based) phosphoproteomics analysis, we identified a large network of cell-intrinsic alterations in signaling in T2D. Over 300 phosphosites showed impaired or reduced insulin signaling, including losses in the classical insulin-stimulated PI3K/AKT cascade and their downstream targets. In addition, we identified over 500 phosphosites of emergent, i.e., new or enhanced, signaling. These occurred on proteins involved in the Rho-GTPase pathway, RNA metabolism, vesicle trafficking, and chromatin modification. Kinome analysis indicated that the impaired phosphorylation sites represented reduced actions of AKT2/3, PKCθ, CHK2, PHKG2, and/or STK32C kinases. By contrast, the emergent phosphorylation sites were predicted to be mediated by increased action of the Rho-associated kinases 1 and 2 (ROCK1/2), mammalian STE20-like protein kinase 4 (MST4), and/or branched-chain α-ketoacid dehydrogenase kinase (BCKDK). Inhibiting ROCK1/2 activity in T2D induced pluripotent stem cell–derived hepatocytes restored some of the alterations in insulin action. Thus, insulin resistance in the liver in T2D did not simply involve a loss of canonical insulin signaling but the also appearance of new phosphorylations representing a change in the balance of multiple kinases. Together, these led to altered insulin action in the liver and identified important targets for the therapy of hepatic insulin resistance.

## Abstract 2

Utilizing patient-derived human inducible pluripotent stem cells differentiated into hepatocytes and phosphoproteomic analysis, we have identified a large network of cell-intrinsic alterations in signaling in type 2 diabetes.

## Graphical abstract

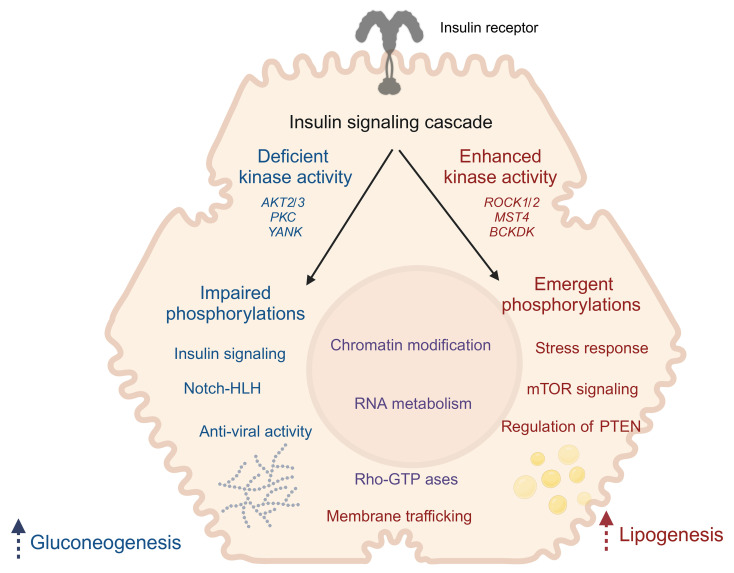

## Introduction

Insulin resistance is central to type 2 diabetes (T2D), obesity, and metabolic syndrome (1–3) and is a significant risk factor for hyperlipidemia, cardiovascular disease, and metabolic dysfunction–associated steatotic liver disease (MASLD) (4–8). The importance of hepatic insulin resistance is emphasized by the bidirectional relationship between MASLD and T2D, such that hepatic steatosis increases the risk of T2D, and insulin resistance increases the risk of MASLD, steatohepatitis, and liver cirrhosis (9–12). At a molecular and cellular level, insulin resistance in the liver is characterized by an altered ability of insulin to stimulate its receptor and downstream kinases (13, 14), resulting in an inability of insulin to suppress hepatic glucose production and glycogenolysis. In most individuals, however, insulin retains its ability to stimulate lipid synthesis, resulting in hepatic steatosis. This differential in glucose and lipid responsiveness has been termed “selective insulin resistance” (15), although to what extent this is due to differences in insulin action at a molecular level, modification of these pathways by circulating factors, or to differential responses to the hyperinsulinemia associated with T2D and metabolic syndrome remains a matter of debate (16, 17).

Studies in animal models and, to a lesser extent, humans have suggested that various circulating factors may contribute to hepatic insulin resistance. For example, overnutrition and obesity can increase circulating lipid levels, leading to ectopic lipid accumulation in muscle and liver. A variety of these lipid species, including diacylglycerides (DAGs), free fatty acids (FFAs), sphingolipids, and ceramides, can contribute to insulin resistance by stimulating various serine/threonine kinases, including members of the novel PKC family, resulting in increased serine/threonine phosphorylation of the insulin receptor (IR) substrates IRS1 and IRS2, the IR, and other proteins, which collectively lead to decreased insulin action (18, 19). Obesity and T2D are also associated with hyperinsulinemia and increases in circulating cytokines, branched chain amino acids (BCAAs), and other metabolites, which can, directly and indirectly, affect liver metabolism by altering insulin signaling and substrate delivery to the liver (20–22). At the individual patient level, T2D is driven by multiple genetic and epigenetic factors, which can contribute to cell-intrinsic insulin resistance and extrinsic factors. The importance of intrinsic factors is evident in the fact that insulin resistance precedes and predicts the development of T2D (2). Identifying the molecular nature of these intrinsic alterations, however, is difficult, since liver tissue or hepatocytes are not easily obtained (23), and when studied in vivo, insulin signaling and metabolic homeostasis in hepatocytes can be altered by circulating factors such as cytokines and metabolites, as well as adjoining stellate cells, endothelial cells, and circulating inflammatory cells.

We have previously shown that induced pluripotent stem (iPS) cells taken from individuals who have T2D or who have insulin resistance but without T2D, when differentiated into myoblasts, demonstrate an insulin resistance signature in vitro, indicating the ability of these cells to model the cell-intrinsic defects in insulin signaling in T2D and insulin resistance (24–26). As noted above, however, alterations in insulin action and metabolism in other issues, especially the liver, are also important in both glucose and lipid homeostasis. To understand the molecular defects underlying hepatocyte insulin resistance, in the present study, we developed a human iPS cell–derived hepatocyte (iHep) model and used this to define the alterations in basal and insulin-regulated phosphorylation using liquid chromatography with tandem mass spectrometry–based (LC-MS/MS–based) global phosphoproteomics in control individuals and patients with T2D. Here, we show that iHeps from patients with T2D had altered insulin signaling of 2 distinct types. The first was represented by a loss of normal insulin regulation of phosphorylation, i.e., impaired signaling. These alterations were present in the canonical insulin signaling pathway and some pathways related to Rho-GTPases and gene transcription. The second class of alterations was represented by gains in the regulation of phosphorylation, i.e., emergent signaling. These alterations included a second distinct subset of proteins in the Rho-GTPase pathway and proteins involved in RNA metabolism, vesicle trafficking, and chromatin modification. Using a recently developed kinase prediction model, we demonstrate that the impaired changes in T2D phosphorylation mapped to pathways regulated by AKT2 and potential changes in PKCθ, CHK2, PHKG2, and STK32C. In contrast, the emergent phosphorylation events are predicted targets of Rho-associated kinases 1 and 2 (ROCK1/2), mammalian STE20-like protein kinase 4 (MST4), and branched-chain α-ketoacid dehydrogenase kinase (BCKDK). Indeed, inhibition of ROCK1/2 reversed some of the changes observed in the deficient pathways. These findings define 2 distinct networks of cell-intrinsic alterations in phosphorylation in hepatic insulin resistance in T2D and identify the potential kinases involved. This opens the pathway for independently targeting these 2 types of defects for the treatment and prevention of T2D and insulin resistance–associated liver disease.

## Results

### Directed differentiation of patient-derived inducible hepatocytes.

**Figure 1:**
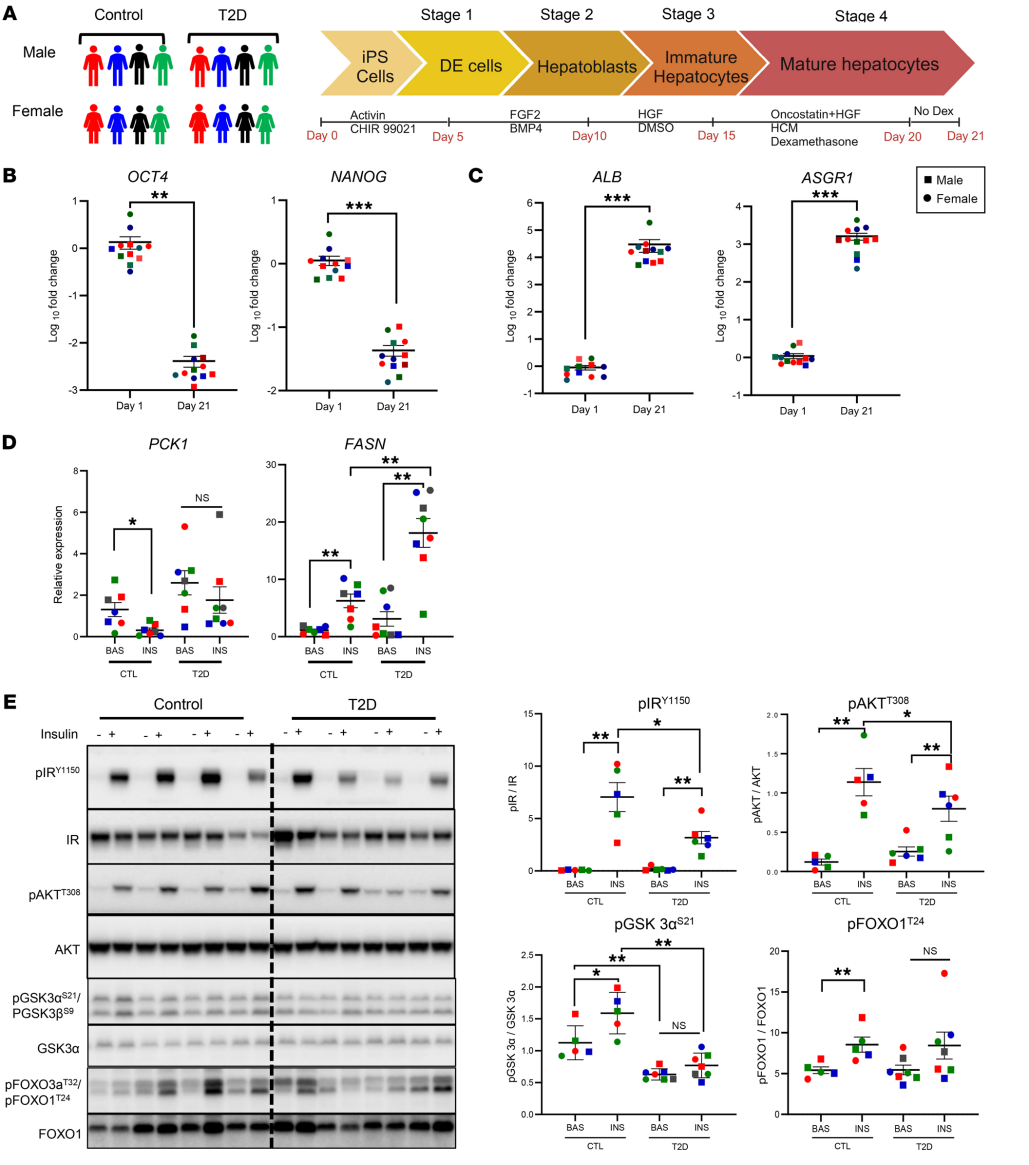
Directed differentiation of iHeps. (**A**) Schematic of iPS cell differentiation into iHeps using a 4-stage growth factor protocol. The cells were obtained from 8 control individuals and 8 patients with T2D. In the **B**–**E**, the cells from male donors are represented by squares and cells from female donors by circles. (**B** and **C**) Gene expression levels of *OCT4*, *NANOG*, *ALB*, and *ASGR1* were determined using RT-qPCR and are plotted on a log_10_ scale. *P < 0.05, **P < 0.01, and ***P < 0.001, for day 1 versus day 21, by paired, 2-tailed *t* test. (**D**) Relative gene expression levels of *PCK1* and *FASN* were determined by RT-qPCR. *n* = 6–8. *P < 0.05 and **P < 0.01, for basal versus insulin, by paired, 2-tailed *t* test and unpaired, 2-tailed *t* test for control plus insulin versus T2D plus insulin. (**E**) Representative immunoblot analysis of phosphorylation of proteins in cells from 4 control and 4 T2D male donors. Quantification of the data in the bar graphs was normalized to total protein on the right. All data indicate the mean ± SEM. CTL, control; BAS, basal; INS, insulin.

To identify cell-intrinsic determinants of insulin resistance in the liver, iPS cells were derived from a cohort of 16 individuals that included 8 patients with T2D and 8 age-matched controls, with equal numbers of males and females. As previously described, the iPS cells were created using Sendai virus reprogramming (Figure 1A). Compared with the control donors, the T2D donor group had a higher average BMI (29.7 ± 0.9 versus 26.8 ± 0.6 kg/m^2^), serum glucose levels (7.5 ± 0.4 versus 5.3 ± 0.1 mM), and hemoglobin A1c (5.7 ± 0.4% versus 4.5 ± 0.1%) (24). The iPS cells were differentiated into hepatocytes (iHeps) over 21 days using the 4-stage growth factor protocol described in Methods (Figure 1A) (27). The differentiated iHeps from both the controls and T2D donors exhibited an almost complete loss of the pluripotency markers OCT4 and NANOG accompanied by simultaneous 10^3^-fold to 10^5^-fold increases in the hepatocyte marker proteins asialoglycoprotein receptor 1 (*ASGR1*), albumin (*ALB*), transthyretin (*TTR*), apolipoprotein A2 (*APOA2*), alpha-fetoprotein (*AFP*), and cytochrome P450 and C2C9 (*CYP2C9*) as determined by quantitative reverse transcription PCR (qRT-PCR) (Figure 1, B and C, and Supplemental Figure 1A; supplemental material available online with this article; https://doi.org/10.1172/JCI183513DS1). Consistent with normal differentiation, at days 8 and 13, there were also transient increases in expression of the hepatocyte stage–specific transcription factors *GATA4* and *HNF4*, which mark hepatic endoderm and immature hepatocytes (Supplemental Figure 1B). Expression of albumin at the protein level was confirmed by immunoblotting and showed a greater than 100-fold increase in iHeps compared with iPS cells, with over 70% of cells strongly positive for albumin, as determined by flow cytometry. This was also confirmed by immunofluorescence (Supplemental Figure 1, C–E).

### T2D iHeps show selective insulin resistance and cell-intrinsic insulin signaling defects.

In addition to gene expression features of normal hepatocytes, control iHeps showed robust gene expression responses to insulin stimulation. Thus, following 3 hours of stimulation with insulin, we detected a 75% decrease in mRNA levels for the key gluconeogenic enzyme phosphoenolpyruvate carboxykinase (*PCK1*) and a 5-fold increase in the levels of mRNA for fatty acid synthase (*FASN*), a key lipogenic enzyme (Figure 1D). Interestingly, in T2D iHeps, insulin-mediated suppression of *PCK1* was lost, while both basal and insulin-stimulated expression of *FASN* was increased almost 2-fold compared with the controls (Figure 1D). Thus, T2D iHeps showed evidence of selective insulin resistance in gene expression, such that insulin did not suppress gluconeogenesis normally but had an exaggerated response on de novo lipogenesis.

To identify potential signaling defects in T2D, fully differentiated iHeps from patients with T2D and from controls were stimulated with 100 nM insulin for 10 minutes. Since glucocorticoids can induce insulin resistance, dexamethasone and hydrocortisone were removed from the growth media 48 hours before insulin stimulation. Western blot analysis showed a classic insulin response in control iHeps with an approximately 90-fold increase in phosphorylation of the IR at tyrosine 1150 (pIR^Y1150^), and this was reduced by approximately 50% in the T2D cells (*P* < 0.05) (Figure 1E). Similarly, AKT^T308^ showed a greater than 9-fold stimulation by insulin in control cells, which was reduced in T2D iHeps by approximately 30% (*P* < 0.05). T2D iHeps also showed lower insulin-stimulated phosphorylation of GSK3α^S21^/GSK3β^S9^ and FOXO1^T24^/FOXO3a^T32^. These changes in phosphorylation occurred with no change in the total protein levels of any of these signaling proteins (Figure 1E). Thus, control iHeps showed robust insulin responses in early insulin signaling, and these events were significantly decreased in cells from patients with T2D in vitro. This occurred in the absence of extrinsic factors that might contribute to insulin resistance, i.e., the changes were cell intrinsic.

### Defining the insulin-regulated phosphoproteome of iHeps.

**Figure 2:**
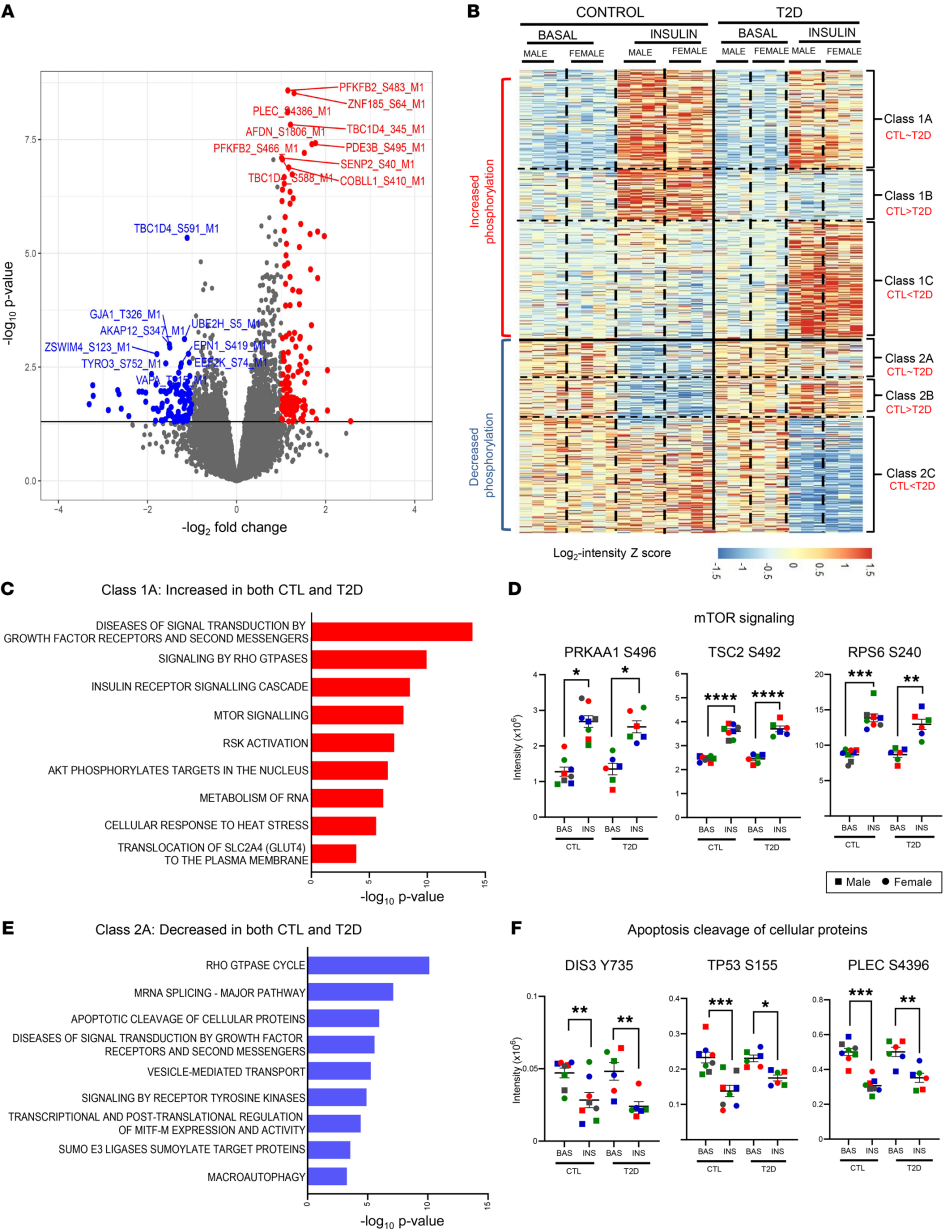
Insulin-regulated phosphosites in control and T2D iHeps. (**A**) Volcano plot showing the phosphopeptides increased or decreased in phosphorylation upon insulin stimulation in control iHeps. (**B**) Hierarchical clustering of phosphosites identified in control and T2D iHeps shows increased and decreased phosphorylation following stimulation with 100 nM insulin for 10 minutes. Rows represent *z* scores of log_2_-transformed intensity of phosphosites for each sample labeled in each column. Classes 1A and 2A show equal increases in both control and T2D cells; classes 1B and 2B show impaired signaling in T2D cells; classes 1C and 2C show emergent signaling in T2D iHeps. (**C** and **E**) Enrichment analysis of overrepresented Reactome pathways analysis of proteins that showed increased or decreased phosphorylation in both control and T2D cells. (**D** and **F**) Quantification of selected phosphosites representing some of the significantly enriched pathways is shown with data from males and females combined. In the panels, the cells from male donors are represented by squares, and female donors are represented by circles, with colors indicating individual donors. *n* = 8 control donors; *n* = 6 T2D donors. *P < 0.05, **P < 0.01, ***P < 0.001, and ****P < 0.0001, by paired, 2-tailed *t* test analysis of raw intensities between groups for basal versus insulin and unpaired and 2-tailed *t* test between control versus T2D with or without insulin. Data are presented as the mean ± SEM.

To more fully define the spectrum of signaling changes, we performed global phosphoproteomics analysis of control and T2D iHeps with and without insulin stimulation (100 nM, 10 minutes) using LC-MS/MS. This identified 46,422 phosphosites, of which 21,863 were considered class I, i.e., had a localization probability of 75% or higher (in these samples, the average was 97%). The fold change of each phosphosite between basal and insulin-stimulated conditions (insulin/basal) in control iHeps is shown as a volcano plot in Figure 2A and by hierarchical clustering (Supplemental Figure 2A). This revealed 292 phosphosites on 226 proteins that increased phosphorylation in response to insulin and 114 phosphosites on 98 proteins that decreased phosphorylation by insulin stimulation (Supplemental Figure 2A and Supplemental Table 1).

Reactome pathway analysis of proteins that increased phosphorylation following insulin stimulation showed enrichment of phosphoproteins related to classical insulin signaling pathways (growth factor signal transduction, IRS-mediated signaling, and mTOR-mediated signaling), as well as programmed cell death, cell junction organization, and RNA metabolism (Supplemental Figure 2B). These phosphosites included many previously described in insulin signaling, including IRS2^S639^, AKT2^S474^, GSK3α^S21^, GSK3β^S9^, ACLY^S455^, pP70S6K^S447^, EIF4B^S422^, MAPK1^Y187^, and MAPK3^Y204^ (Supplemental Figure 2C and Supplemental Table 1), along with some less-studied phosphorylation events on classical signaling proteins, including IRS2^T350,S346,T520^, FOXO1^S319,S329^, FOXO3^S59,T307,S315^, and TSC2^S492,S534^ (Supplemental Table 1). This insulin-stimulated cluster was also enriched in proteins involved in the Rho GTPase cycle, a pathway well known to be insulin stimulated in muscle and adipose tissue (28), but less studied in hepatocytes. This included insulin-stimulated phosphorylation of multiple Rho guanine nucleotide exchange factors (GEFs), such as ARHGEF12^S1288^, which catalyze the exchange of GDP to GTP, as well as phosphorylation on several Rho GTPase-activating proteins (GAPs), such as RALGAPA2^T532^, which act to enhance GTP hydrolysis to GDP, thus maintaining the GTPases in an inactive state (28) (Supplemental Figure 2D and Supplemental Table 1). Among the insulin-regulated phosphorylations in control iHeps, those with the highest fold stimulation were PFKFB2^S466,S483^, SENP2^S40^, PDE3B^S495^, and AFDN^S1806^ (Supplemental Table 1). Previous studies have shown that AKT mediates phosphorylation of PFKFB2^S466,S483^ and plays a vital role in insulin’s effect to increase glycolysis (29, 30). Insulin-stimulated phosphorylation of actin/Rap1-binding protein afadin (AFDN^S1806^) has also been observed in iPS-derived myoblasts (24). Although the functional effect of this phosphorylation is unknown, AKT-mediated phosphorylation of AFDN^S1718^ is thought to play a role in nuclear localization and negative feedback on insulin action by recruitment of histone deacetylase 6 (HDAC6) (31, 32).

Many proteins also showed decreased phosphorylation in response to insulin, as exemplified by acetyl-CoA carboxylase α (ACACA^S25^), spectrin α, nonerythrocytic 1 (SPTAN1^S1029^), tumor protein P53 (TP53^S155^), and poly(ADP-ribose) polymerase 1 (PARP1^T88^) (Supplemental Figure 2, E and F, and Supplemental Table 1). Reactome pathway analysis of these proteins showed enrichment of Rho GTPase cycle effectors (AKAP12^S347^), as well as other proteins involved in vesicle transport (EPN1^S419,S489^), membrane trafficking (RABEP1^S410^, TBC1D4^S588^), SUMOylation (PARP1^T88^, TP53^S155^), and RNA modification (RPL34^S12^), including core components of the spliceosome and several serine/arginine–rich (SR-rich) proteins linked to mRNA splicing and miRNA metabolism (SF3B2^Y379^, SRRM1^S211,T411,S409^, and SRRM2^S919,T1453,T316^) (Supplemental Table 1). Multiple sites on NHERF1^S269,S280,S290,T293^ were also dephosphorylated upon insulin stimulation. NHERF1 (also known as SLC9A3R1) is a sodium/hydrogen exchange regulatory cofactor that can interact with phosphatase and tensin (PTEN) and has been suggested to function as a negative regulator in the InsR/PI3K/AKT1 pathway (33–35). Thus, there was a strong enrichment of classical insulin signaling proteins and many novel proteins whose phosphorylation was either positively or negatively regulated by insulin in control iHeps (Supplemental Table 1).

### Multiple layers of dysregulated insulin signaling in T2D.

To identify the nature of the cell-intrinsic insulin signaling defects in the liver in T2D, we compared the phosphoproteome of control and T2D iHeps with and without insulin stimulation. Principal component analysis (PCA) indicated a clear separation of samples based on insulin stimulation, the presence or absence of T2D, and donor sex (Supplemental Figure 3A). Considering only sex-common sites that exhibited an increase or decrease in phosphorylation upon insulin stimulation in control or T2D iHeps with a *P* value of less than 0.05 and a ±1.5-fold change, 1,007 phosphosites exhibited increases in phosphorylation and 703 sites exhibited decreases in phosphorylation upon insulin stimulation in either the control iHeps, T2D iHeps, or both (Supplemental Figure 2B and Supplemental Table 2). We termed the upregulated phosphosites class 1 and the downregulated phosphosites class 2. Both the insulin-upregulated and insulin-downregulated classes could then be subdivided into sites for which stimulation was similar in control and T2D cells (classes 1A and 2A, representing 362 and 169 sites, respectively). These were (a) sites for which the effect of stimulation was significantly (*P* < 0.05) reduced in T2D cells compared with control cells, i.e., exhibiting “impaired” insulin signaling (classes 1B and 2B), representing 196 and 128 sites, respectively, and (b) sites for which insulin stimulation was significantly (*P* < 0.05) greater in iHeps from patients with T2D than in those from control individuals, which we termed “emergent” signaling (classes 1C and 2C), representing 429 and 426 sites, respectively (Figure 2B and Supplemental Table 2), of which 249 and 302 met the criteria of both showing ±1.5-fold stimulation and a *P* value of less than 0.05 in the insulin-stimulated state.

Focusing on class 1A phosphosites, Reactome pathway analysis showed enrichment of proteins involved in signal transduction by growth factors, mTOR signaling, IRS-mediated signaling, Rho GTPase cycle, and metabolism of RNA that were not significantly altered by T2D, despite the presence of insulin resistance. These included insulin stimulation of PRKAA1^S496^, TSC2^S492^, and RPS6^S240^ (Figure 2, C and D, and Supplemental Table 2). On the other hand, class 2A phosphosites, i.e., those decreased by insulin to a similar extent in both control and T2D cells, were enriched in proteins involved in the Rho GTPase cycle, mRNA splicing pathway, SUMOylation, and the apoptosis pathway. The latter included phosphorylation of DIS3^Y735^, TP53^S155^, and PLEC^S4396^ (Figure 2, E and F). DIS3 is a 3′ to 5′ exoribonuclease and catalytic subunit of the exosome, a protein complex involved in the degradation and processing of both nuclear and cytoplasmic RNA species, and PLEC encodes plectin, a cytoskeletal protein that maintains tissue integrity.

### Impaired insulin signaling in T2D iHeps.

**Figure 3:**
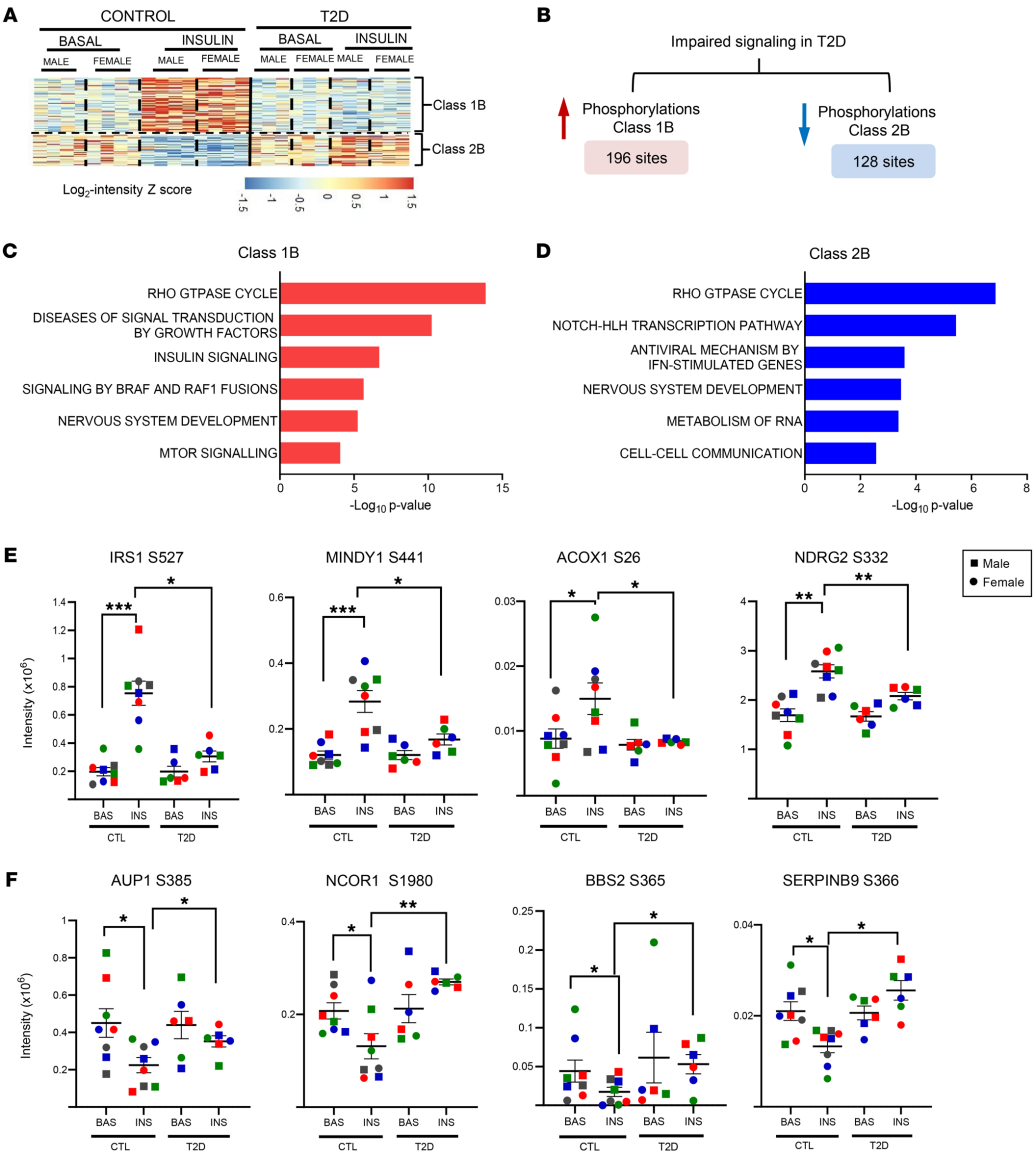
Impaired insulin signaling in T2D iHeps. (**A**) Hierarchical clustering showing impaired insulin-regulated phosphosites in control and T2D iHeps. Rows represent normalized log intensity *z* scores for intensities of the phosphosites for each sample. (**B**) Schematic showing the number of phosphosites with impaired signaling in T2D iHeps, i.e., phosphorylation events that were lost or had significantly reduced regulation in T2D compared with control cells. Data were separated into phosphosites normally increased by insulin stimulation (left) and sites normally decreased by insulin (right), using data shown in the heatmap in Figure 2B. (**C** and **D**) Reactome pathway enrichment of “impaired sites” in T2D iHeps. (**E** and **F**) Quantification of exemplary impaired phosphosites normally increased phosphorylation in **E** or decreased phosphorylation in **F**. In the panels, the cells from male donors are represented by squares, and those from female donors are represented by circles, with colors indicating individual donors. *n* = 8 controls; *n* = 6 T2D. *P < 0.05, **P < 0.01, ***P < 0.001, and ****P < 0.0001, by paired, 2-tailed *t* test analysis of raw intensities between groups for basal versus insulin, and unpaired, 2-tailed *t* test between control versus T2D with or without insulin. Data are presented as the mean ± SEM.

More important in the context of insulin resistance are the phosphosites altered by T2D. Class 1B phosphosites showed reduced phosphorylation following insulin stimulation in iHeps from patients with T2D compared with those from control individuals, indicating “impaired” insulin signaling (Figure 3, A and B, and Supplemental Table 3). This group included many important components of the IR/IGF1R and Rho-GTPase pathways (Figure 3, C and E). Among these, IRS1^S527^, a site ascribed to S6 kinase phosphorylation, showed impaired signaling in T2D cells. Interestingly, this may be a liver-specific alteration, since this site shows no alteration in phosphorylation in skeletal muscle or iPS cell–derived myoblasts from patients with T2D as compared with controls (24, 36). In addition, ACOX1^S26^ and NDRG2^S332^ show impaired signaling in T2D. Acyl-CoA oxidase 1 (ACOX1) is involved in FA metabolism by peroxisomes, and phosphorylation at ACOX1^S26^ protects from polyubiquitination and proteasomal degradation, thereby increasing activity. NDRG2^S332^ phosphorylation, on the other hand, can be mediated by AKT and novel protein kinase Cs (PKCs) and is also decreased in lipid-induced insulin resistance in C2C12 cells (37). This is thought to play a role in the inactivation of PTEN (38). MINDY^S441^ (also known as FAM63A) also shows reduced insulin-stimulated phosphorylation in T2D. MINDY is a Lys-48 deubiquitinase, and intronic and missense variants of MINDY1 have been associated with T2D, suggesting that normal MINDY function is protective of T2D (39). In the present study, the signal transduction proteins EIF4B, RPS6, RPS6KB1, and TSC2 also showed impaired phosphorylation in T2D, as did ERBIN, MARK3, and several proteins involved in Rho GTPase signaling, including the Rho GAPs ARHGAP32, Rho GEFs (ARHGEF7, ARHGEF11), and the Rho effector FARP1 (Supplemental Figure 3, B and C, and Supplemental Table 3).

Class 2B phosphosites, on the other hand, represent sites whose phosphorylation was decreased by insulin stimulation in controls, and this response was impaired in T2D iHeps. This subclass was enriched in proteins not classically associated with insulin signaling but involved in the Notch/HLH transcription pathway, antiviral mechanisms of IFN-stimulated genes, RNA metabolism, and the Rho-GTPase cycle (Figure 3, D and F). For example, insulin stimulation reduced the phosphorylation of NCOR1^S1980^ in control iHeps, and this effect was lost in T2D (Supplemental Figure 3, D and E). Although the role of this phosphorylation is unknown, phosphorylation of NCOR1^S1460^ decreases its ability to interact with the liver X receptor (LXR), thus increasing the transcription of lipogenic LXR in the liver (40). Also in the class 2B subclass were phosphorylation of AUP1^S385^, BBS2^S365^, and Serpin B9^S366^, all of which were decreased by insulin stimulation and showed reduced insulin regulation in T2D. Ancient ubiquitous protein 1 (AUP1) plays an important role in lipid droplet formation, degradation on INSIG1, and SREBF1 and ApoB stability (41–43). BBS2 is a ciliary protein involved in the pathogenesis of Bardet-Biedl syndrome (BBS), a genetically heterogeneous disease characterized by obesity, diabetes, and hyperphagia. Genetic knockout of BBS2 in mice results in hepatic insulin resistance (44–46). Other phosphorylations normally downregulated by insulin that were impaired in the T2D cells included the proteins in the Rho GTPase cycle, albeit these involved different phosphosites and proteins than did those falling into class 1B (Supplemental Table 3).

### Emergent insulin signaling in T2D iHeps.

**Figure 4:**
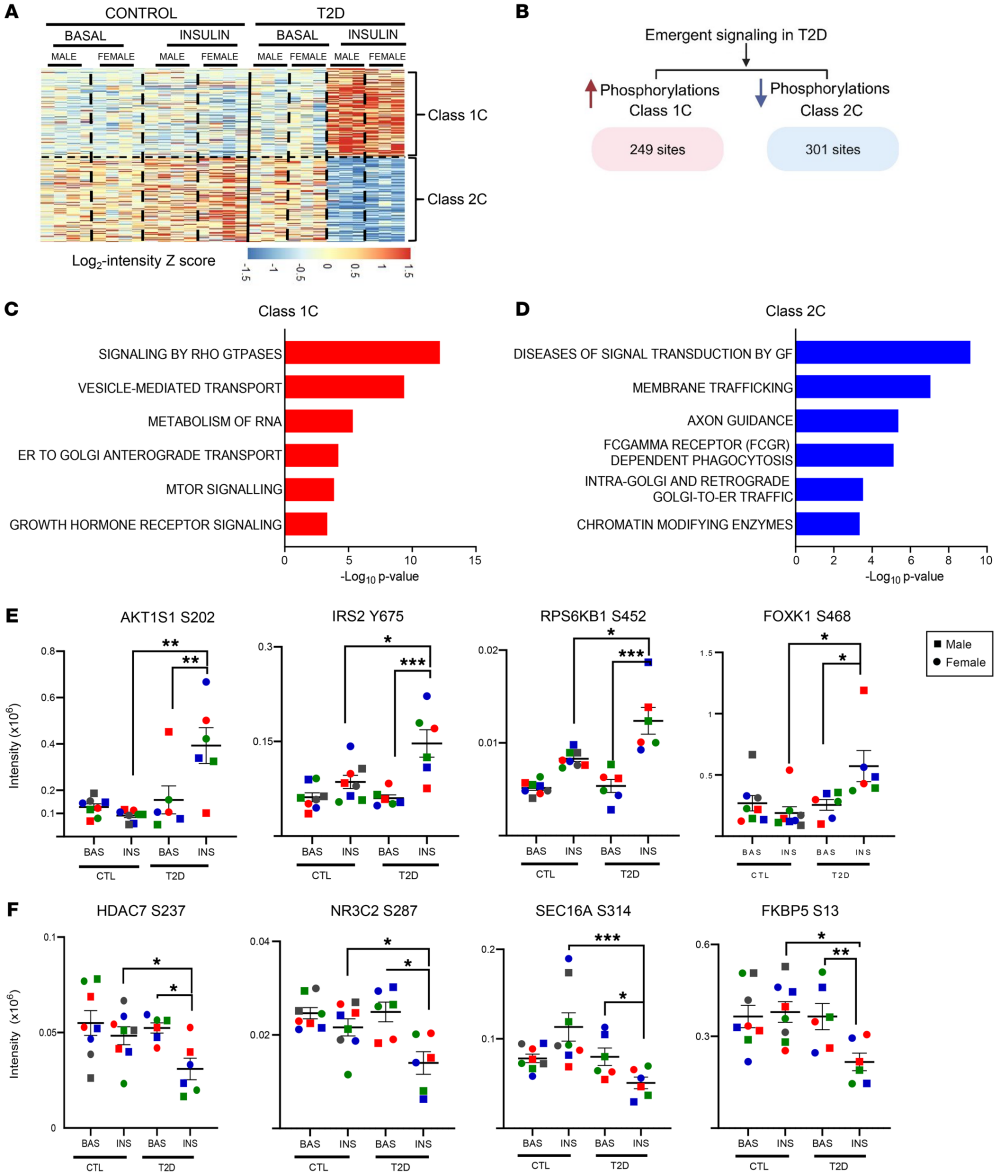
Emergent insulin signaling in T2D iHeps upon insulin stimulation. (**A**) Hierarchical clustering showing insulin-regulated phosphosites in control and T2D iHeps. Rows represent normalized *z* scores corresponding phosphosite intensities for each sample. (**B**) Schematic showing the number of phosphosites with “emergent” signaling in T2D cells, i.e., phosphorylation events that were increased or had significantly reduced regulation in T2D compared with control cells using data from the heatmap in Figure 2B. (**C** and **D**) Reactome pathway enrichment of “emergent sites” in T2D iHeps. (**E** and **F**) Quantification of exemplary emergent phosphosites normally increased in phosphorylation in **C** or decreased in phosphorylation in **D**. In the panels, the cells from male donors are represented by squares and those from female donors by circles, and colors indicate individual donors. Data are presented as the mean ± SEM. *n* = 8 control; *n* = 6 T2D. *P < 0.05, **P < 0.01, and ***P < 0.001, by paired, 2-tailed *t* test of raw intensities between groups for basal versus insulin, and unpaired, 2-tailed *t* test between control versus T2D with or without insulin.

The most interesting classes of altered protein phosphorylations in T2D are classes 1C and 2C. These phosphosites represent subsets of insulin-regulated phosphorylations that showed little or no response to insulin in control cells but markedly increased or decreased their phosphorylation in T2D iHeps. Thus, these sites represent emergent or new insulin-regulated phosphorylations in the context of insulin resistance (Figure 4, A and B).

Class 1C phosphosites, i.e., those that were increased by insulin stimulation in T2D iHeps by greater than 1.5-fold and with a *P* value of less than 0.05 but with little or no change in control cells, were enriched for phosphosites on a third subset of proteins involved in Rho-GTPase signaling but different from those in classes 1B and 2B, which showed impaired or defective phosphorylation, as well as enrichment for proteins involved in vesicle-mediated transport, metabolism of RNA, mTOR signaling, and regulation of PTEN (Figure 4, C and E). These phosphosites included increased phosphorylation of AKT1S1^S202^, which increased its association with the scaffold protein 14-3-3, thereby decreasing the pool of free AKT1S1 and lowering the activation of mTORC1 (47, 48). Interestingly, phosphorylation on IRS2^Y675^, a direct substrate of the IR and a residue in a PI3K-binding motif (49), was enhanced in T2D iHeps. RPS6KB1^S452^, a protein involved in mTOR signaling and whose phosphorylation is increased by insulin stimulation, also showed increased phosphorylation in T2D cells, as did phosphorylation of FOXK1^S468^, a transcription factor involved in insulin action that can physically interact with IRs (50) and play a role in the regulation of the gluconeogenic genes *PCK1* and *G6Pc* in the liver (51). Other proteins involved in vesicle-mediated transport also showed emergent signaling in T2D and are summarized in Supplemental Figure 4, A and B, and Supplemental Table 4.

Finally, class 2C represents emergent phosphosites for which there was little response to insulin in control cells but whose phosphorylation was significantly reduced upon insulin stimulation in T2D cells (Figure 4, D and F). This included another subset of proteins in the membrane trafficking pathway and proteins in pathways of retrograde Golgi transport, such as ARID1A^S1754,S1755^, which were dephosphorylated in response to insulin in T2D iHeps but not in control cells (Supplemental Table 4). Deletion of ARID1A in liver has been shown to increase susceptibility to the development of hepatic steatosis and insulin resistance by impairing FA oxidation (52). Dephosphorylation of SEC16A^S314^, a protein involved in ER-to-Golgi transport (53, 54) in T2D, as well as the mineralocorticoid receptor NR3C2^S283,S387^ are sites known to affect receptor stability (55). NR3C2 has also been found to affect glucose regulation in skeletal muscle (56). FKBP5^S13^, which plays a major role in regulating AKT1activity, also showed emergent dephosphorylation. Finally, several proteins involved in chromatin modification, such as EP300, EP400, and KMT2D, showed insulin-stimulated decreases in phosphorylation in T2D iHeps, which was not seen in controls (Supplemental Figure 4, C and D). The role of these chromatin-modifying proteins in insulin signaling and T2D has yet to be explored.

### Basal phosphorylation defects in T2D.

**Figure 5:**
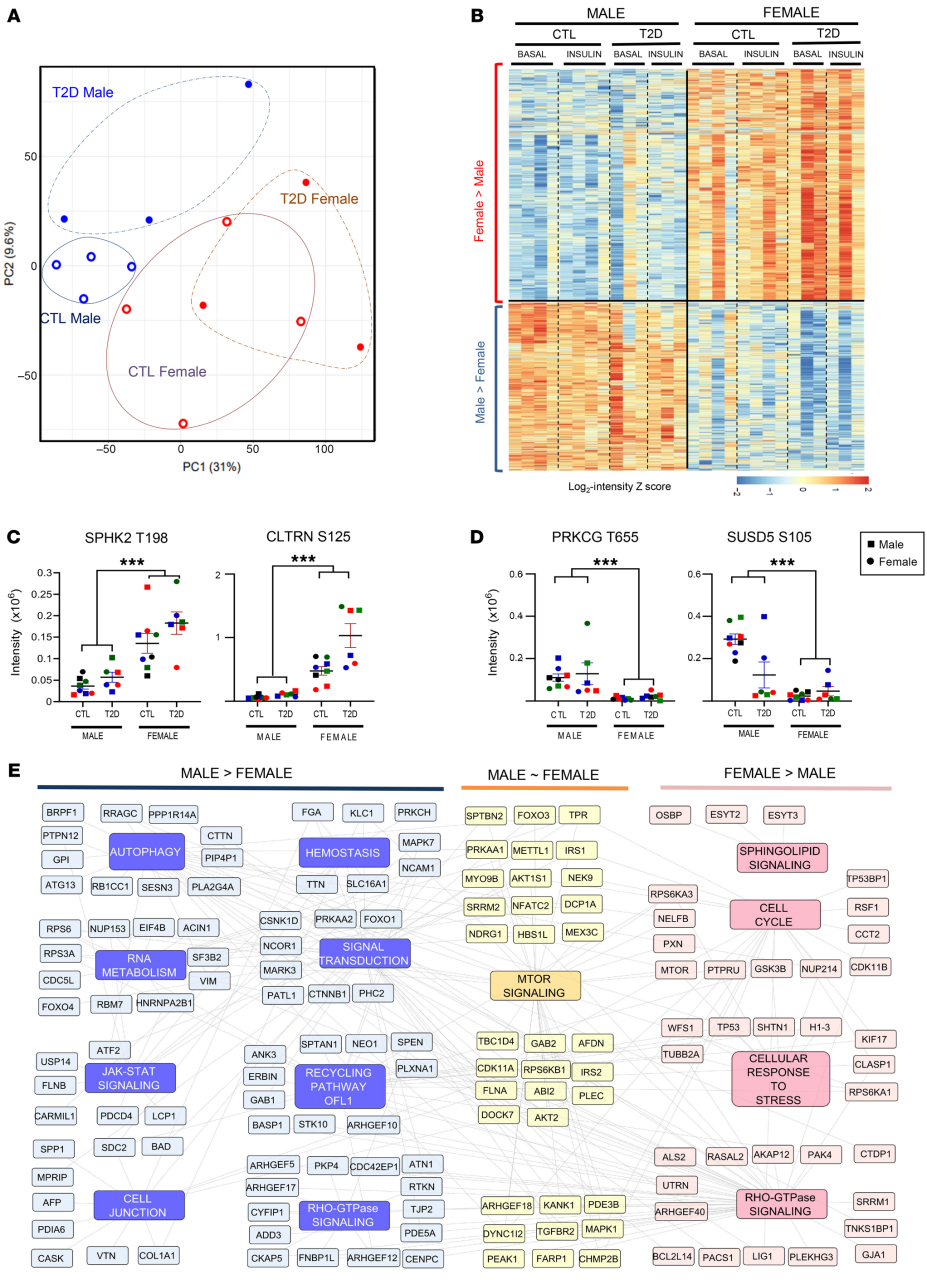
Sexual dimorphism in the basal and insulin-stimulated phosphoproteome. (**A**) PCA plot showing separation of the phosphoproteome after adjusting for the surrogate variable by phenotype (control, open circles; T2D, closed circles) and sex (blue, males; red, females) in the basal state. (**B**) Hierarchical clustering of phosphopeptides after adjusting for the surrogate variable that shows sexual dimorphism (F-test FDR <5%). Rows represent *z* scores of log_2_-transformed intensity of phosphosites for each sample in each column. (**C** and **D**) Quantification of basal and insulin-stimulated phosphorylation levels of exemplary proteins showing sexual dimorphism. In the panels, the cells from male donors are represented by squares, and those from female donors are represented by circles, with colors indicating individual donors. (**E**) Signaling map representation of some of the most enriched biological pathways and related proteins that exhibited significantly higher phosphorylation ratios in males (blue) versus females (pink). Data are presented as the mean ± SEM, ***P < 0.001, based on the F-test for sex from unadjusted intensities.

In addition to differences in insulin-regulated phosphorylations, PCA of the phosphoproteomics data revealed alterations in basal phosphorylation between control and T2D iHeps (Figure 5A). Pathway analysis of these proteins showed enrichment in many of the same pathways exhibiting alterations in the insulin-regulated state. Thus, we observed increased basal phosphorylation in proteins involved in the Rho GTPase cycle, vesicle-mediated transport, cell-cell communication, diseases of signal transduction, and processing of mRNA (Supplemental Figure 5A and Supplemental Table 5), whereas proteins showing lower basal phosphorylation were enriched in pathways involved in the Rho-GTPase cycle, mRNA splicing, and IFN induced with helicase C domain 1–mediated (IFIH1-mediated) induction of IFN-α (Supplemental Figure 5B and Supplemental Table 5). Many proteins in the insulin signaling pathway, including IRS2^S391,T527,T350,S1100^, FOXO1^S287,S329^, GSK3B^T390^, and PIK3R1^Y467^, also exhibited significantly lower phosphorylation in the basal state in T2D iHeps (Supplemental Figure 5C). Proteins that exhibited higher basal phosphorylation in T2D iHeps were enriched in the Rho-GTPase cycle, including CKAP4^S460^, RANBP2^S2626^, and PPP1R12A^S422^ (Supplemental Figure 6A). By contrast, basal phosphorylation of CREBBP^S2364^, PCBP2^S188^, and TNAIP2^S645^, which are involved in IFIH1-mediated induction of IFN-α/-β antiinflammatory proteins, was lower in T2D cells (Supplemental Figure 6B). Protein kinase N1 (PKN1^T774^) also showed significantly increased basal phosphorylation, a site linked to PKN1 activation, in T2D iHeps (Supplemental Figure 6C). PNK1 has been implicated in adipocyte differentiation, insulin-induced actin cytoskeleton reorganization, and GLUT4 translocation (57) and is also identified as an insulin-regulated kinase in iHeps in the kinome analysis described below.

### Identification of sex-specific phosphoproteomic changes in iHeps.

A patient’s sex can be a modifier of disease pathogenesis, and both T2D and MASLD occur more frequently in men than women (58). In previous studies, we observed that the sex of the donor modifies the phosphoproteome of iPS cell–derived myoblasts (iMyos) (25). This was also true in iHeps. Indeed, of the 21,863 sites quantitated, 2,680 sites (12.2%) showed sexual dimorphism, with 1,553 sites exhibiting female dominance and 1,127 showing male dominance using an FDR of less than 0.1 (Figure 5, A and B, and Supplemental Table 6). Among the female-dominant sites were those on the amino acid transporter collectrin (CLTRN^S125^) and on the sphingosine kinase SPHK2^T198^ (Figure 5C). Consistent with this, previous studies have shown that knockout of SPHK2 improves insulin sensitivity in male mice but not female mice (59). Male-dominant phosphosites, on the other hand, included protein kinase C γ (PRKCG^T635^) and Sushi domain–containing 5 protein (SUSD5^S105^) (Figure 5D). The latter has been implicated in insulin resistance in adipose tissue (60). We also observed sexual dimorphism in both control and T2D iHeps upon insulin stimulation (Supplemental Figure 6D). Pathway analysis revealed male-dominant insulin-stimulated phosphorylation differences related to RNA metabolism, autophagy, and signal transduction proteins, whereas female-dominant phosphorylations occurred in pathways related to the cell cycle, cellular response to stress, and sphingolipid metabolism. A network diagram with representative proteins showing sex differential phosphorylation is shown in Figure 5E.

### Defining the kinases that contribute to normal and altered insulin signaling in iHeps.

Defining the potential protein kinases involved in the altered insulin action of T2D is a challenge, since the exact kinases responsible for many altered phosphorylation sites are unknown, and many kinase-substrate relationships remain undefined. Recently, Johnson et al. developed a tool to deconvolute these relationships using synthetic peptide libraries to profile the substrate sequence specificity of 303 mammalian Ser/Thr kinases, representing more than 84% of the Ser/Thr kinases present in human cells (61). These include many kinases not previously linked to insulin action. Probing the specific amino acid sequences surrounding the phosphoserines and phosphothreonines in our phosphoproteomics data against this database, we sought to identify the kinases involved in insulin signaling in iHeps and those that are altered in activity in T2D by ranking the top S/T kinases predicted to target that phosphorylation site and creating a percentile score, as previously described (61). In agreement with the known insulin signaling cascade, when this algorithm was applied to identify the kinases contributing to insulin regulation of phosphorylation in control iHeps, the top kinases predicted to be involved (at >95% confidence) were members of the AKT family (AKT1, AKT2); the S6 kinase family (P70S6K, RSK3, p90RSK); serum glucocorticoid kinase 3 (SGK3); and the phosphorylase kinases PHKG1 and PHKG2. This analysis also identified some less-well-studied kinases, including protein kinase N (PKN1, -2, and -3), which has been linked to insulin stimulation of GLUT4 translocation in adipocytes (57), and the doublecortin kinases DCAML1 and DCAML2, which have also recently been linked to insulin action (62) (Supplemental Figure 7A and Supplemental Table 7).

**Figure 6:**
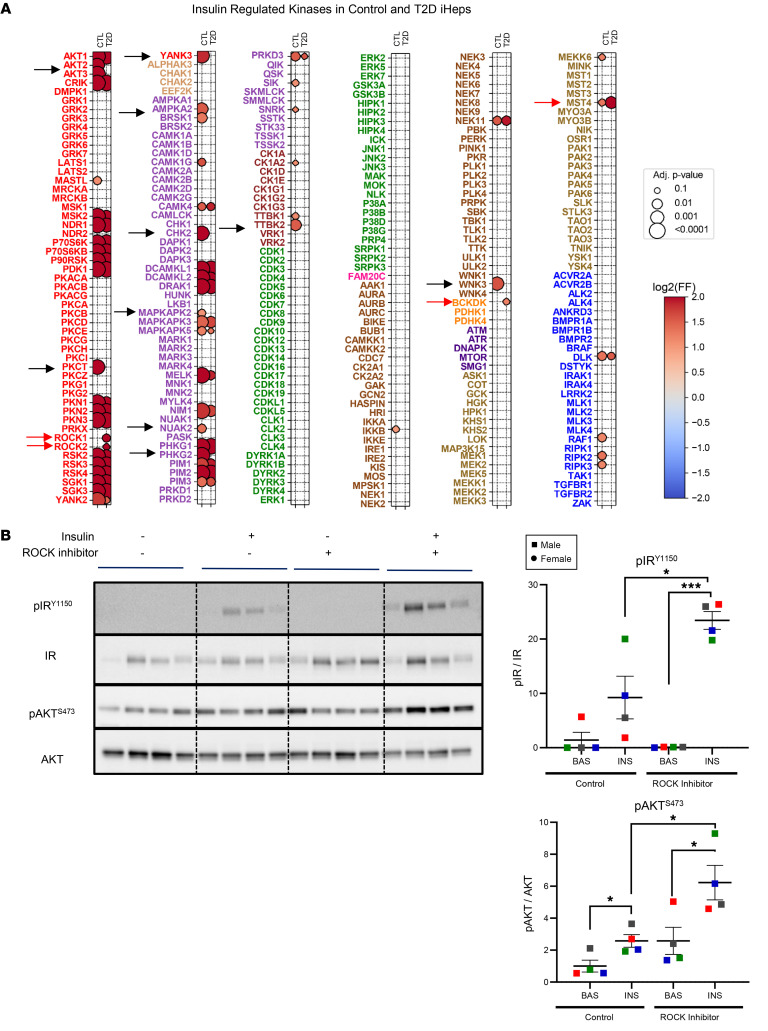
Kinome profiling of predicted kinases related to insulin stimulation and altered in T2D. (**A**) Bubble plot showing kinases predicted to be activated in iHeps upon insulin stimulation for control and T2D cells. In the bubble maps, the size and color represent the adjusted *P* values and frequency factor (FF), respectively, for the significant kinases (adjusted *P* ≤ 0.1). The black arrows indicate kinases with reduced activity in T2D following insulin stimulation, and the red arrows show the kinase predicted to have increased activity in T2D upon insulin stimulation. (**B**) Representative immunoblot analysis of phosphorylation of proteins in 4 T2D male donors with and without 100 nM insulin and 10 nM ripasudil. Quantifying the data in bar graphs normalized to a total protein is on the right. In the panels, the cells from male donors are represented by squares, and color indicates individual donors. Data are presented as the mean ± SEM. *n* = 6–8. *P < 0.05 and ***P < 0.001, by 2-tailed, paired, 2-tailed *t* test for basal versus insulin, insulin versus insulin+inhibitor, and unpaired, 2-tailed *t* test for insulin versus inhibitor.

More interestingly, the kinome analysis allowed a direct comparison of the kinases predicted to contribute to the altered serine/threonine phosphorylation in T2D in the insulin-stimulated states. This is shown as a bubble plot in Figure 6A comparing kinases activated in control versus T2D cells. Consistent with the above data and with previous studies, kinome analysis predicted that reduced activity of the kinase family member AKT2 was contributing to the reduced insulin-stimulated phosphorylations in T2D. The kinome analysis also predicted reduced activity of several other kinases, including PKCθ, YANK3 (STK32c), AMPKA2, BRSK2, MAPKAPK2, CHK2, PHKG2, TTBK2, and WNK3, as contributors to the impaired insulin-stimulated phosphorylations observed in T2D iHeps (Figure 6A, highlighted with black arrows). By contrast, for the substrates showing increased phosphorylation in T2D iHeps, kinome analysis best mapped to increased activity of the Rho-associated kinases ROCK1 and -2, as well as BCKDK and MST4 (Figure 6A, highlighted with red arrows). A summary of the altered substrate phosphorylations predicted to be the result of these increases or decreases in kinase activity is provided in Supplemental Table 8, and the top altered impaired or emergent kinase-substrate pairs in T2D are shown in Supplemental Figure 7B.

For those kinases detected in the LC-MS/MS proteomics analysis, none of the kinases with predicted reduced or increased activity showed a difference in abundance between control and T2D iHeps at the protein level, except CHK2, which was higher at the protein level, but predicted to be lower in activity in T2D compared with control iHeps, suggesting that the changes in activity were not likely due to changes in kinase abundance (Supplemental Figure 8). On the other hand, several of the predicted alterations were consistent with previous studies. Thus, ROCK1 protein levels and activity are increased in the liver of mice on a high-fat diet (HFD) (63), BCAA metabolism has been linked to insulin resistance and T2D (20), and the STE20-type kinases MST3 and MST4 have been linked to the progression of hepatocellular carcinoma (64). To directly test whether increased ROCK activity contributes to the altered insulin signaling in T2D, iHeps from control individuals and patients with T2D were treated with a selective inhibitor of ROCK1 and -2, ripasudil (63, 65). Proximal insulin signaling assessed by immunoblotting showed significant improvement by this treatment (Figure 6B and Supplemental Figure 9), indicating that the overactivity of ROCK kinases likely contributes to the intrinsic alterations in insulin signaling observed in T2D iHeps.

### An integrated picture of the insulin-regulated phosphoproteome in normal and T2D iHeps.

**Figure 7:**
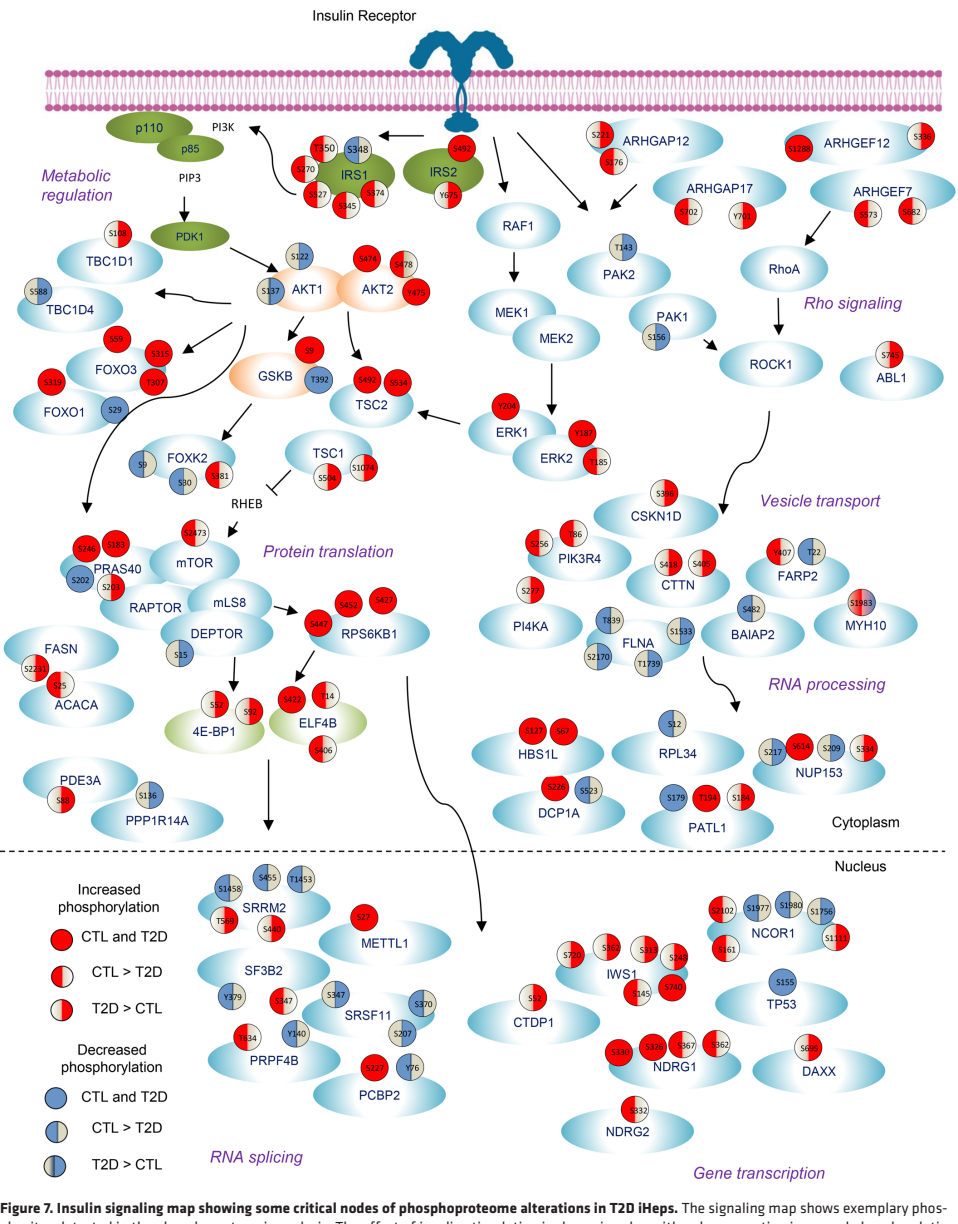
Insulin signaling map showing some critical nodes of phosphoproteome alterations in T2D iHeps. The signaling map shows exemplary phosphosites detected in the phosphoproteomic analysis. The effect of insulin stimulation is shown in color, with red representing increased phosphorylation and blue representing decreased phosphorylation (a significant difference at *P* < 0.05). For each site, the stimulation (up or down) in the control iHeps is indicated in the left half of the circle, while that for the T2D iHeps is indicated in the right half of the circle. Each of the phosphosites is color coded according to the effects of insulin on phosphorylation. The pathways shown are representative of those enriched in the Reactome pathways analysis.

Putting all of the above data together, we can construct a composite insulin-signaling map highlighting some of the important alterations in protein phosphorylation in T2D iHeps, including impaired and emergent changes (Figure 7). Note that in the proximal insulin-signaling pathway, insulin-dependent phosphorylation of AKT2^S474^ was preserved in T2D, whereas phosphorylation of AKT2^S478^ was reduced. AKT2^S478^ is less well explored in insulin signaling. It has been reported that Cdk2/cyclin A can phosphorylate AKT2^S478^ and synergize with S474 phosphorylation at the HM site to activate this kinase allosterically (66). Interestingly, although serine phosphorylation of IRS1 is usually regarded as being negatively regulatory for insulin signaling (36), multiple sites of serine phosphorylation of IRS1 were reduced in T2D iHeps compared with controls, suggesting that these are part of a normal feedback mechanism and that this mechanism may be lost in T2D. In addition, we observed reduced phosphorylation of mTOR^S2473^ and reduced phosphorylation of multiple proteins involved in Rho signaling, including ARHGAP12^S176^, ARHGEF7^S572,S608^ and ARGAP17^S702^.

The emergent sites of Ser/Thr phosphorylation in T2D iHeps were equally prominent. These include TSC1^S504,S1074^, 4E-BP1^S52,S92^, and multiple sites on the transcription elongation factor IWS1. TSC1 is an important negative regulator of mTOR signaling, and loss of TSC1 results in mTOR-dependent increased phosphorylation of the ribosomal protein S6, p70S6K, and 4E-BP1, leading to cell growth and proliferation. IWS1, an RNA processing regulator recruits the histone methyltransferase SETD2 to RNAPII and regulates the splicing of FGFR2-related genes (67). Some proteins had multiple alterations. The nuclear corepressor NCOR1 showed losses in the regulation of sites that in iHeps from control patients showed both increased and decreased phosphorylation following insulin stimulation. This could potentially modify its ability to interact differentially with and alter the activity of its different transcription factor partners, including LXR, ERR, and PPAR. Likewise, closely related proteins sometimes showed changes in opposite directions. For example, in T2D iHeps, IRS1 were found to have many sites that lost insulin-stimulated serine phosphorylation, whereas IRS2 had sites that gained phosphorylation. Likewise, phosphorylation of TBC1D4^S588^ was reduced in T2D iHeps, while phosphorylation of TBC1D1^S108^ was increased.

Finally, it is worth noting that multiple alterations in phosphorylation were observed for proteins in the nucleus, including proteins involved in transcription (IWS1, NCOR1, and NDRG1) and RNA splicing (PCBP2, PRPF4B, SF3B2, and SRRM2). IWS1 showed multiple sites of emergent phosphorylation in T2D cells (IWS1^S248,S313,S362,S720^), whereas phosphorylation of NDRG1^S362,S367^ after insulin stimulation was lost. Likewise, among insulin downregulated sites, NCOR1^S1756,1977^ phosphosites were lost in T2D cells. Interestingly, 3 proteins involved in RNA splicing lost insulin regulation of sites of tyrosine phosphorylation normally downregulated following insulin stimulation, including PCBP2^Y46^, PRPF4B^Y140^, and SF3B2^Y379^, suggesting loss of action of a nuclear tyrosine kinase or gain of action of a nuclear tyrosine phosphatase in T2D cells. SRRM2, on the other hand, lost 3 insulin-downregulated phosphosites (SRRM2^S455,S2453,S1458^) and gained 2 insulin-upregulated sites (SRRM2^S440,T569^). SRRM2 organizes splicing condensates to regulate alternative splicing, however, the effects of these phosphorylations on this activity remain to be determined.

## Discussion

Hepatic insulin resistance is central to the pathogenesis of T2D, fatty liver disease, and their metabolic complications (4, 8). Dissecting insulin action and the molecular mechanisms underlying insulin resistance in the liver, however, is very challenging, since access to tissue in humans is limited, and, in vivo, the liver is subject to the effects of changing levels of multiple hormones and growth factors that may modify insulin signaling, as well as to the effects related to inter-tissue transfer of substrates contributing to the overall physiological responses (68). To overcome these challenges and define the cellular alterations contributing to insulin resistance in liver in humans with T2D, in the present study we developed a new model using iPS cells derived from patients with T2D differentiated in vitro into hepatocytes (iHeps). These cells exhibited multiple markers of well-differentiated hepatocytes, including high expression levels of *ALB* and *ASGR1*, and allowed insulin to inhibit the expression of *PCK1* while stimulating the expression of *FASN*. In addition, insulin stimulated the phosphorylation of ACLY^S455^, a known target of AKT and PKA, and phosphorylation of this site increases the enzymatic activity of ACLY by 6-fold (69). Likewise, insulin stimulated the phosphorylation of PRAS40^S203,S183,T246^, with S183 and T246 being well-studied sites of insulin action through AKT and mTORC1 (70). On the other hand, acetyl-CoA carboxylase 1 (ACC1), a lipogenic enzyme that is activated by insulin, was dephosphorylated at S25 upon insulin stimulation (71). Insulin also regulates the phosphorylation of multiple proteins belonging to the Rho GTPase pathway, including ARHGEF7 and ARHGEF18, which are increased by insulin, and AKAP12 and YES1, which are decreased in phosphorylation by insulin.

Using the iHep model, we have defined the insulin signaling defects in hepatocytes in human T2D. These defects were cell intrinsic, i.e., observed in vitro in the absence of any differences in circulating factors that occur in diabetes. These cell-intrinsic defects include alterations in the classical insulin signaling pathway and many alterations beyond this pathway (summarized in Figure 7). Using LC-MS/MS, we were able to quantitate nearly 22,000 phosphorylation events in the basal and insulin-stimulated states, and almost 900 (~4%) were altered in the T2D iHeps. Of these, 37% of sites exhibited “impaired” signaling, i.e., loss of a normal insulin-stimulated event, representing the classical concept of insulin resistance and 63% exhibited “emergent” signaling, i.e., an appearance of insulin-regulated phosphorylations not observed or significantly greater than those observed in control cells. Thus, insulin resistance was characterized as much or more by gains of phosphorylation as by losses of phosphorylation.

The impaired phosphorylation events included many sites in the canonical insulin signaling pathway. Since most of these phosphorylations are involved in the positive regulation of these insulin signaling intermediates, reduced phosphorylation levels would be predicted to reduce the activity of these proteins and enzymes and their downstream effects. Although serine phosphorylation of IRS1 is usually regarded as being negatively regulatory for insulin signaling (36), somewhat surprisingly, insulin-stimulated phosphorylation of IRS1, for example IRS1^S527^, was decreased in the T2D iHeps. This suggests that these phosphorylations were part of a normal feedback mechanism and that this mechanism was also lost in the insulin resistance of T2D. Interestingly, MINDY^S441^ phosphorylation was lost in T2D upon insulin stimulation, and MINDY gene variants are associated with an increased risk of T2D (39). Impaired signaling also included impaired phosphorylation of sites that normally decrease phosphorylation levels upon insulin stimulation and showed significantly less reduction in T2D iHeps. These included sites on AUP1 and BBS2, two proteins that are important in hepatic lipogenesis (41–45).

Although less frequently discussed, but more numerous and potentially more interesting, are the alterations we have identified as emergent signaling in insulin resistance. Pathway analysis revealed enrichment of proteins involved in Rho-GTPase signaling, RNA metabolism, mTOR signaling, and chromatin-modifying enzymes. Rho family members play roles in many processes, including endocytosis of LDL receptors (72). These emergent changes also included the appearance of increased phosphorylation of AKT1S1, RPS6KB1, and FOXK1 in T2D cells. Interestingly, the mineralocorticoid receptor (MR), NR3C2, and FKBP5 showed emergent phosphorylations in T2D cells, suggesting crosstalk between insulin and steroid hormone signaling in hepatocytes in T2D (55) and providing new potential sites for therapeutic intervention.

While most of the alterations observed in T2D are present in cells from both male and female donors, it is important to note that a significant percentage of phosphorylation events, both in the basal and insulin-stimulated states, showed marked differences dependent on the sex of the donor. We have previously observed sex-based differences in protein phosphorylation in iPS cell–derived myoblasts from control individuals and patients with T2D (24), as well as insulin-resistant and insulin-sensitive nondiabetic individuals (25), and others have reported this in human skeletal muscle in vivo (73). It is known that males have a slightly higher prevalence of T2D (74), more obesity-related hepatic insulin resistance (75), and a higher prevalence of MASLD (76). This has been traditionally described as the effects of sex hormones. However, these iHep studies were done in vitro without added sex hormones, indicating that these differences were cell intrinsic. While determining the mechanisms underlying these differences will require further study, these sex-based differences may be pathophysiologically important. The quantitative data for SPHK2^T198^ and CLTRN^S125^, in which phosphoproteomics showed 3- to 4-fold high levels of phosphorylation in cells of females than in males, and for PRKCG^T655^ and SUSD5^S105^, for which the opposite was true. The phosphorylation of SPHK2^T198^ was strongly female dominant. While the role of this specific site of phosphorylation remains to be determined, ERK1 is known to phosphorylate SPHK2^S351,T578^. Interestingly, knockout of SPHK2 improves insulin sensitivity in male, but not female, mice (55). Other studies have shown sexual dimorphism in the phosphoproteome in both rodents (77) and humans (78).

One unique aspect of the present study is the application of a newly developed atlas of substrate specificities (61) and computational modeling to identify the kinases potentially responsible for the alterations in phosphorylation in iHeps from patients with T2D. Using the sequences surrounding the individual protein phosphorylation sites in our phosphoproteomics dataset and this atlas of the preferred substrate motifs for more than 300 known serine/threonine kinases in the human kinome, we can predict the kinases most likely to be linked to altered phosphorylation in T2D. Consistent with the known canonical insulin signaling cascade, in control iHeps, this algorithm identified many of the kinases involved in insulin signaling, including members of the AKT, RSK, and S6K families. It also identified the kinases that contribute to the reduced insulin-stimulated phosphorylations in T2D, including a reduction in the activity of AKT2, as well as reductions in AMPKA2, MAPKAPK2, PHKG2, and some less-studied kinases with regard to insulin action, including YANK3/STK32c, BRSK2, TTBK2, and WNK3. The kinome analysis of T2D iHeps also predicted the kinases responsible for emergent signaling that may act as drivers of insulin resistance, including ROCK1/2, BCKDK, and MST4 for insulin-regulated phosphorylations. Indeed, we found that treatment of human T2D iHeps with a selective ROCK1/2 inhibitor could restore dysregulated proximal insulin signaling, suggesting that these may be some of the driving kinases in cell-intrinsic insulin resistance. This was also suggested by other studies showing that ROCK1 activity is higher in the liver of humans with metabolic dysfunction–associated steatotic liver disease (MASLD) (79), and hepatocyte-specific ROCK1 knockout inhibits hepatic de novo lipogenesis in rodents (75). Treatment with ROCK inhibitors and genetic ablation of ROCK1 have also been shown to ameliorate inflammation and fibrosis in rodent models of NASH, as well as hepatic steatosis and insulin resistance (79, 80). BCKDK and MST4 are also predicted to increase activity in T2D iHeps by kinome analysis and have been linked to the regulation of lipid droplet dynamics and metabolic stress (81). Thus, emergent phosphorylations may contribute to the enhanced lipogenesis observed in T2D. At the same time, the impaired signaling, e.g., decreased AKT activity, may be more closely linked to the loss of regulation of gluconeogenesis, creating a pattern of “selective insulin resistance.” In vivo, chronic hyperinsulinemia and altered substrate supply from overnutrition may also play significant roles in these divergent responses (82), but the differential response of iHeps from T2D patients to insulin action on *PCK1* and *FASN* expression indicates that at least part of this difference in response is cell intrinsic. Further studies of these cell-intrinsic alterations with circulating factors will be needed to dissect these differences completely.

In summary, using iPS cell–derived human hepatocytes from patients with T2D, we have defined defects in phosphorylation-mediated signaling in T2D in vitro and without the interference of exogenous circulating factors. This revealed a complex network of cell-intrinsic alterations, including changes in the canonical insulin signaling pathway and in Rho GTPase signaling, Notch signaling, RNA metabolism, vesicle transport, and mTOR signaling. These alterations manifested as decreases in insulin’s effect on many phosphosites normally upregulated or downregulated by insulin stimulation, as well as the appearance of new, emergent insulin-regulated increases or decreases in specific phosphorylation events. Analysis of these alterations predicted that these changes were due to losses in activity of AKT2, YANK3, and/or PKCθ and to increases in ROCK1/2, MST4, and/or BCKDK activity. By defining these alterations in phosphorylation and the dysregulation of the kinases potentially responsible for these changes, we have not only gained better insight into the cellular basis of insulin resistance in the liver in T2D but have also identified new therapeutic targets for the treatment of T2D.

## Methods

### Sex as a biological variable.

The iPS cells were derived from 8 individuals with T2D and 8 control individuals, with equal numbers of males and females.

Additional details on methods are available in the Supplemental Methods.

### iPS cell culturing and differentiation into iHeps.

The iPS cells used for hepatocyte differentiation were originally derived from skeletal muscle biopsies performed at the Karolinska Institute using an approved protocol and informed consent, as previously described (24). The iPS cells were maintained in mTESR1 medium (STEMCELL Technologies). For hepatocyte differentiation, confluent iPS cells (passages 16–18) were treated with 100 ng/mL activin A and 3 μM CHIR99021 for 3 days to induce differentiation into definitive endoderm (stage 1). This was followed by treatment with 5 mg/mL basic FGF-2 (bFGF-2) and 20 ng/mL bone morphogenetic protein BMP4 for 5 days to induce differentiation into the hepatic endoderm (stage 2), followed by treatment with 20 ng/mL hepatocyte growth factor for 5 more days to induce the immature hepatocyte stage (stage 3). Finally, the cells were treated with hepatocyte basal medium supplemented with hepatocyte culture medium (HCM) with 20 ng/mL HGF, 20 ng/mL oncostatin, and 100 nM dexamethasone for 8 days to reach the mature hepatocyte state (stage 4). Dexamethasone and insulin supplementation were removed for the last 48 and 24 hours, respectively, before the signaling experiments. The cells were then left in starvation media overnight before insulin stimulation. All cell lines were studied simultaneously to avoid experimental variation across studies. Sex was considered as an important biological variable in this study and is denoted in most figures and in the data analysis. iPS cells were derived from 8 individuals with T2D and 8 control individuals, with equal numbers of males and females.

### Cell lysate preparation.

The confluent and mature iHeps at day 21 were placed in basal media (0.1% BSA in RPMI) overnight and then stimulated with or without 100 nM insulin for 10 minutes. The cells were washed 3 times with ice-cold TBS, lysed in 4% sodium deoxycholate (SDC) and 100 mM Tris-HCl, pH 8.5, boiled immediately at 95°C for 10 minutes, and then sonicated. Protein content was adjusted to 250 g using the BCA assay. For LC-MS/MS, samples were then reduced with 1 mM tris(2-carboxyethyl)phosphine (TCEP); alkylated with 40 mM 2-chloroacetamide (CAA); digested with trypsin and lysC (1:100, enzyme/protein w/w) overnight; and then loaded on proteomics-RPS cartridges with 300 μL isopropanol with 1% trifluoroacetic acid (TFA) and spun for 10 minutes at 300*g*. Cartridges were washed with 200 μL isopropanol with 1% TFA and spun at 500*g* for 4 minutes, and then washed with 200 μL 0.2% TFA and spun at 500*g* for 4 minutes. Peptides were eluted with 150 μL 80 % acetonitrile (ACN) and 5% ammonium hydroxide. Ten percent of the eluate was taken and dried in a SpeedVac.

### LC-MS/MS measurement.

Dried peptides were solubilized with 105 μL equilibration buffer (80% ACN, 0.1% TFA), and phosphopeptides were enriched using the AssayMap Bravo. Priming buffer (99.9% ACN, 0.1% TFA), elution buffer (1% ammonium hydroxide), and equilibration buffer were used following the standard protocol provided by Agilent Technologies. The enriched phosphopeptides were dried in the SpeedVac, resolubilized in 5 μL 2% ACN and 0.3% TFA, and injected into the mass spectrometer.

Samples were loaded onto 15 cm columns packed in-house with C18 1.9 μM Reprosil particles (Dr. Maisch), with an EASY-nLC 1000 system (Thermo Fisher Scientific) coupled to an Exploris 480 mass spectrometer (Thermo Fisher Scientific). A column oven maintained the column temperature at 60°C. Peptides were introduced onto the column with buffer A (0.1% formic acid) and then eluted with a 120-minute gradient starting at 5% buffer B (80% ACN, 0.1% formic acid) followed by a stepwise increase to 30% in 95 minutes, 60% in 5 minutes, 95% over two 5-minute periods and 5% in 2 × 5 minutes at a flow rate of 300 nL/min. Phosphopeptides were eluted with an 85-minute gradient starting at 3% buffer B (80% ACN, 0.1% formic acid) and followed by a stepwise increase to 19% in 40 minutes, 41% in 20 minutes, 90% in 15 minutes, and 3% in 10 minutes , at a flow rate of 300 nL/min.

A data-independent acquisition MS method was used for proteome and phosphoproteome analyses. For the proteome, 1 full scan (300 to 1,650 *m/z*, *R* = 120,000 at 200 *m/z*) at a target of 3 × 10^6^ ions was initially performed. This was followed by 48 windows with a resolution of 15,000, in which precursor ions were fragmented with higher-energy collisional dissociation (fixed collision energy, 27%) and analyzed with an automatic gain control (AGC) target of 1 × 10^6^ ions in profile mode using positive polarity. For phosphoproteome measurements, 1 full scan (300 to 1,400 *m/z*, *R* = 120,000 at 200 *m/z*) at a target of 3 × 10^6^ ions was first performed, followed by 32 windows with a resolution of 30,000, in which precursor ions were fragmented with higher-energy collisional dissociation (stepped collision energy: 25%, 27.5%, 30%) and analyzed with an AGC target of 3 × 10^6^ ions in profile mode using positive polarity.

### Phosphoproteomics analysis.

The phosphosite intensities were log_2_ transformed, and the transformed data were confirmed to approximately fit a normal distribution. Data were then reviewed for the relationship between the transformed missingness probability and the average of the observed intensity. The missing values were imputed using KNN truncation (KNN-TN) (83), and the samples were normalized to have the same median log_2_ intensity. The variancePartition package (84) was applied to discover the drivers of variation in phosphosite intensities. Surrogate variable (SV) analysis was performed (85), and a single SV was constructed and adjusted for. Two samples from T2D donors (1 male and 1 female) had to be excluded from the final analysis because of a lower state of differentiation and/or poor resolution of the phosphoproteome. To identify the differentially abundant phosphosites among more than 2 groups, the R package limma (86) was used to perform linear modeling and moderated F-tests and to determine *P* values and FDRs (87) with the adjustment of the SV. The clustering of significant phosphosites was performed using an F-test FDR of less than 0.1 and hierarchical cluster analysis based on the Euclidean distance of these selected phosphosites. The phosphosite clusters in the hierarchical dendrogram using a variable cut height approach (88) were identified according to a hierarchical tree. To gain biological insight into this clustering result, overrepresentation analysis using Reactome pathway gene sets was conducted with the tool clusterProfiler (89, 90). Stimulation ratios are presented as log_2_(treated/basal) ratios, in which treated and baseline samples were paired by cell line. For analysis of the sites that were differentially abundant between basal and insulin-stimulated states, linear modeling and moderated *t* tests with adjustment of SV and cell lines (paired tests) were performed. To discover the phosphosites that were differentially abundant between control versus T2D, linear modeling and moderated *t* tests with adjustment of the SV (unpaired tests) were performed.

### Kinome analysis.

The phosphorylation sites detected in this study were scored using the data collected on 303 S/T kinases, and their ranks in the known phosphoproteome score distribution were determined (percentile score) as previously described (61). For every singly phosphorylated site, kinases ranked within the top-15 S/T kinases were considered biochemically predicted kinases for that phosphorylation site. In assessing kinase motif enrichment, we compared the percentage of phosphorylation sites for which each kinase was predicted among the upregulated and downregulated phosphorylation sites between the 2 conditions being compared (FDR <0.5 for basal control versus T2D and FDR <0.25 for control versus T2D treated), versus the percentage of biochemically favored phosphorylation sites for that kinase within the set of unregulated sites in this study (FDR >0.5 for basal control versus T2D and FDR >0.25 for control versus T2D treated). Contingency tables were corrected using Haldane correction. Statistical significance was determined using a 1-sided Fisher’s exact test, and the corresponding *P* values were adjusted using the Benjamini-Hochberg procedure. For every kinase, the most significant enrichment side (upregulated or downregulated) was selected on the basis of the adjusted *P* value and presented in volcano plots and bubble maps. Bubble maps were generated with size and color strength representing the adjusted *P* values and frequency factors, respectively, only displaying significant kinases (adjusted *P* ≤ 0.1).

### Insulin signaling and immunoblot analysis.

The iHeps grown in starvation media (RPMI plus B27 without insulin and growth factors) overnight were stimulated or not with 100 nM insulin for 10 minutes, after which the cells were washed 3 times with ice-cold TBS. For ROCK1/2 inhibition,10 nM ripasudil (Cayman Chemical) was used for 3 hours before insulin stimulation. The cells were then snap-frozen in liquid nitrogen until further analysis. A RIPA buffer was then used to homogenize the cells. Protein estimation was done using a bicinchoninic acid (BCA) assay kit. SDS-PAGE was used to resolve equal protein amounts (10–15 mg), after which proteins were transferred onto PVDF membranes (MilliporeSigma) and immunoblotted with the following commercial antibodies: IR β (Tyr1150/1151) (19H7) rabbit mAb (no. 3024, Cell Signaling Technology [CST]); insulin receptor β (4B8) rabbit (no. 3025, CST); pAKT (Thr308) (D25E6) rabbit mAb (no. 13038, CST); pAKT (Ser473) (D9E) rabbit mAb (no. 4060, CST); pan-AKT (C67E7) rabbit mAb (no. 4691, CST); pGSK-3α (Ser21) (36E9) rabbit mAb (no. 9316, CST); GSK-3α (D80D1) rabbit mAb (no. 4818, CST); pFoxO1 (Thr24)/FoxO3a (Thr32) antibody (no. 9464, CST); FoxO1 (C29H4) rabbit mAb (no. 2880, CST); pPRK1 (Thr774)/PRK2 (Thr816) antibody (no. 2611, CST); PKN1 polyclonal antibody (catalog 720396, Thermo Fisher Scientific); and human serum albumin antibody (catalog MA5-29022, Thermo Fisher Scientific).

### RNA isolation and quantitative PCR.

Total RNA was isolated using TRIzol (Thermo Fisher Scientific), and cDNA was synthesized with 500 ng RNA using a High-Capacity cDNA Reverse Transcription kit (Applied Biosystems). The quantitative PCR (qPCR) reaction was done using iQSYBR Green Supermix (Bio-Rad) on a CFX384 thermocycler (Bio-Rad).

### Statistics.

For immunoblotting and qPCR, the data are presented as the mean ± SEM. Comparisons between 2 groups, i.e., basal versus T2D, without versus with insulin, were performed using an unpaired and paired, 2-tailed Student’s *t* test, respectively. The significance level was set at a *P* value of less than 0.05 unless otherwise indicated. To account for multiple hypothesis testing when relevant, *P* values were adjusted using the Benjamini-Hochberg FDR rate, where an FDR of less than 10% was considered significant. GraphPad Prism 9 (GraphPad Software) was used for graphing, and Cytoscape 3.9.1 and Adobe Illustrator were used for figures.

### Study approval.

These studies were approved by the ethics committee at the Karolinska Institute. Informed consent was obtained from all study participants.

### Data availability.

The log_2_-transformed, imputed, normalized, SV-adjusted data used for analysis are provided in Supplemental Table 9. Each sample is described by the donor phenotype, sex, cell line indicating the individual, and the presence or absence of insulin stimulation, e.g., “CTL_M_C426_Basal.” The code will be available upon request. Values for all data points in graphs are reported in the Supporting Data Values file.

## Author contributions

AKG and CRK designed the research, performed the experiments, analyzed the data, and wrote the manuscript. MT, AKJ, and MM performed the phosphoproteomics experiments. JLJ, TMY, and LCC performed the kinome analysis. JMD and HP performed the bioinformatics analysis. CRK designed the research and supervised the project.

## Supplementary Material

Supplemental data

Unedited blot and gel images

Supplemental table 1

Supplemental table 2

Supplemental table 3

Supplemental table 4

Supplemental table 5

Supplemental table 6

Supplemental table 7

Supplemental table 8

Supplemental table 9

Supporting data values

## Version 1 04/15/2025

Electronic publication
