# Supplementary material for: Cell-intrinsic insulin signaling defects in human iPS cell–derived hepatocytes in type 2 diabetes
Source: J Clin Invest. 2025 Apr 15;135(8):e183513. doi: 10.1172/JCI183513 (PMC11996863; doi:10.1172/JCI183513)
Supplement: Supplemental data [file jci-135-183513-s174.pdf]

Supplementary Figure 1

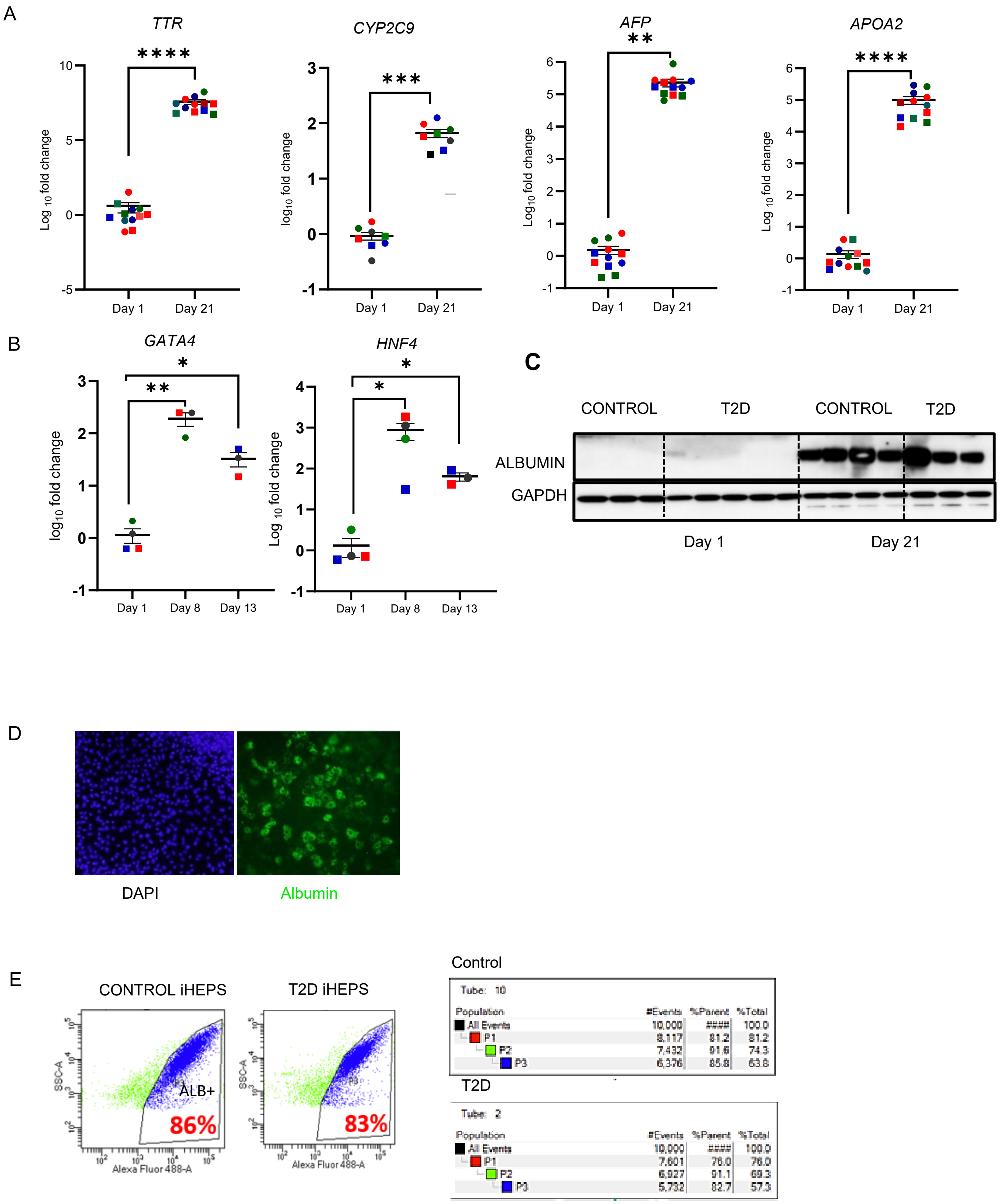

Supplementary Figure S1: Characterization of the differentiated iHeps. A) Gene expression of markers for hepatocyte differentiation and maturity at day 1 and day 21 as determined by RT-PCR (n=6-8 per group). B) Gene expression of intermediate differentiation stage-specific markers at Days 1, 8, and 13 of differentiation were determined by RT-PCR. Cells from male donors are represented by squares and female donors by circles, and color indicates individual donors. Data are shown as mean +/- SEM, n=6-8. Significance is based on paired t-tests between day 1 and day 21. * $P<0.05$, ** $P<0.01$,***$P<0.001$,****$P<0.0001$. C) Immunoblot showing protein expression of albumin in CTL and T2D iHeps at Day 1 and 21, with GAPDH represents a housekeeping protein n=3-4 per group. D) Immunofluorescent staining of iHeps for DAPI staining on the left panel and intracellular albumin (HSA) on the right panel. E) FACS staining of iHeps for albumin indicating the percent of cells showing positivity of ALB+(blue) gated on live cells shown in the inset among the live cells stained with PI (green), and a representative from control and T2D iHep.

Supplementary Figure 2

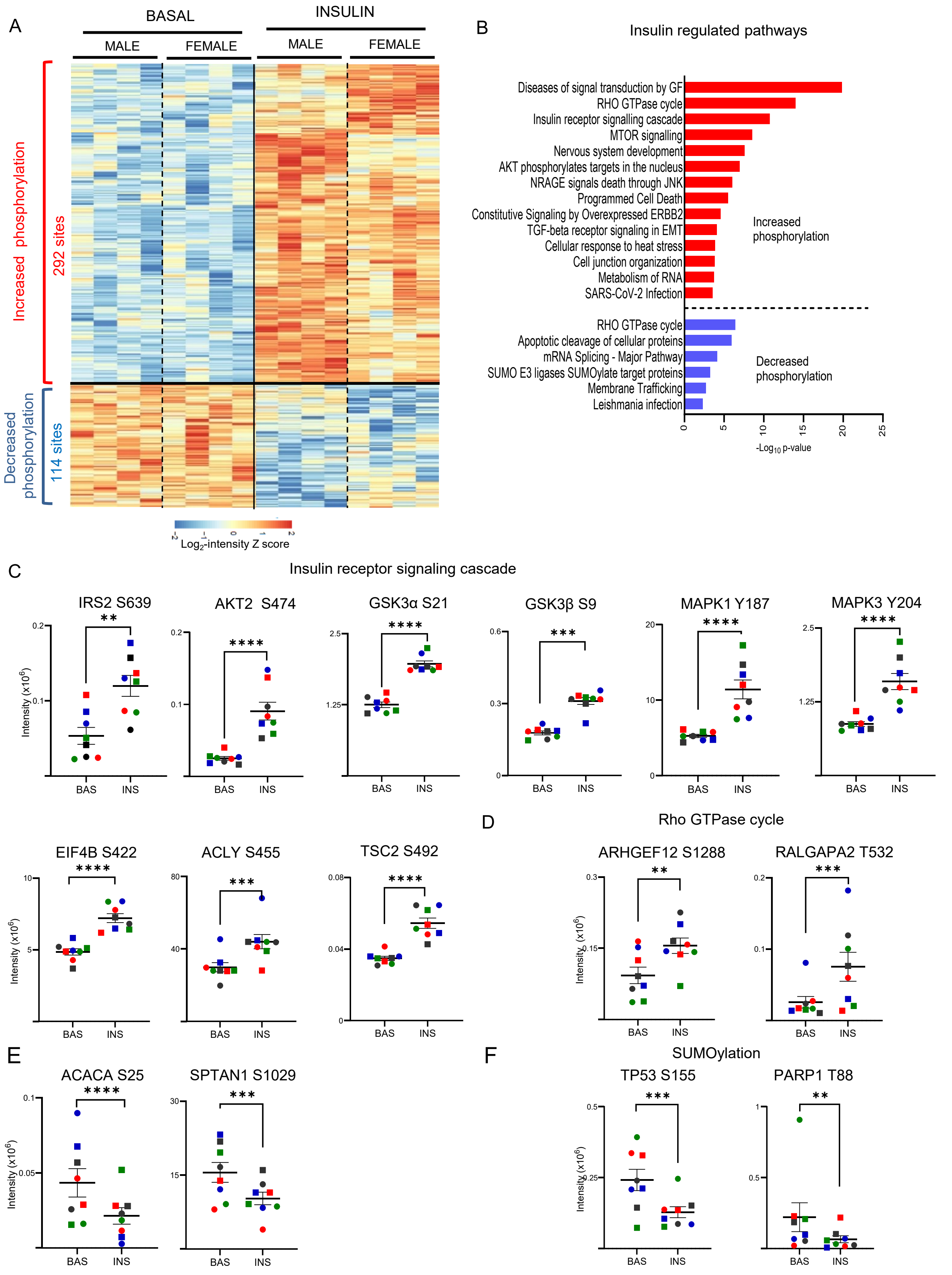

Supplementary Figure S2: Insulin-regulated phosphosites in CTL iHeps. A) Hierarchical clustering of phosphosites showing insulin-regulated sites in control iHeps. Rows represent Z scores of log_2 transformed intensity of phosphosites for each sample labeled in each column. These are subclassified as denoted on the right. B) Enrichment analysis of overrepresented Reactome pathways analysis of proteins that showed increased or decreased phosphorylation in both CTL. The classes correspond to heat map classification. C-F) Selected phosphosites on significantly enriched pathways are shown as examples from the class. Cells from male donors are represented by squares and female donors by circles, and color indicates individual donors. Data are presented as means ± SEM, n=8, * $P<0.05$,** $P<0.01$,***$P<0.001$,****$P<0.0001$, paired t-test analysis of intensities was performed between groups for basal vs insulin. Data from males and females are combined for better representation.

Supplementary Figure 3

A

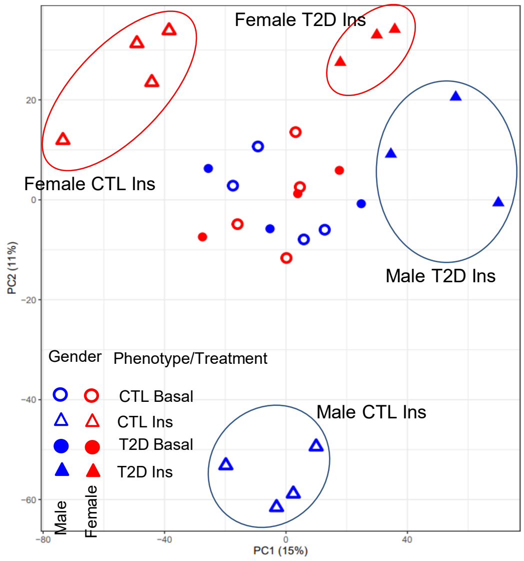

B

Class 1B: Increased phosphorylation and impaired in T2D

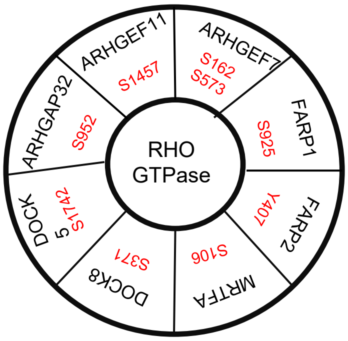

C

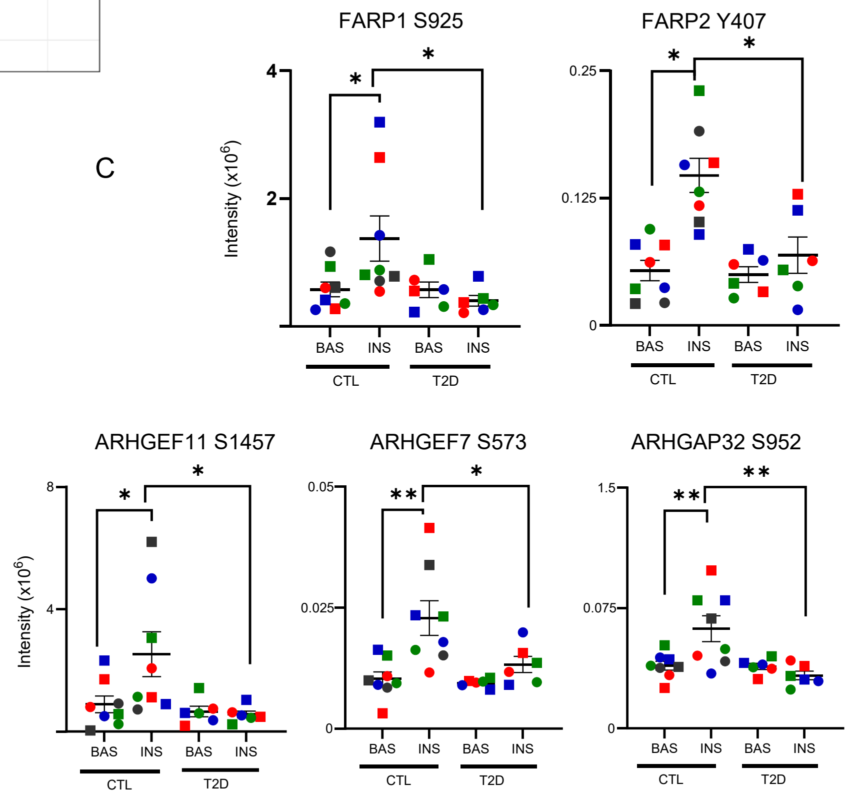

D

Class 2B: Decreased phosphorylation and impaired in T2D

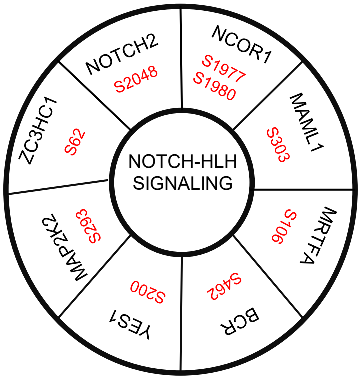

E

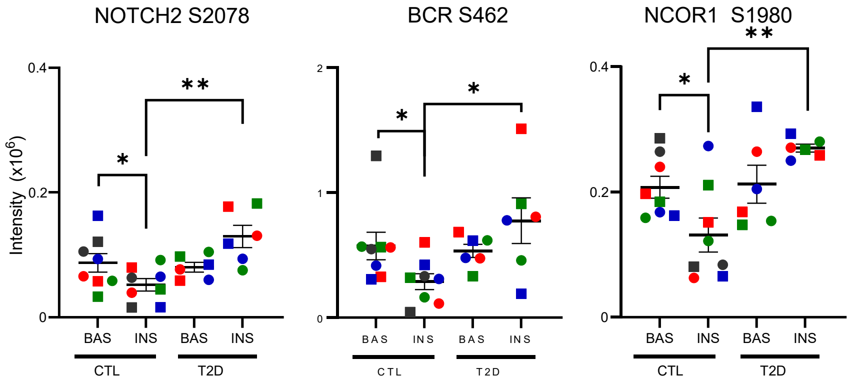

Supplementary Figure S3: Impaired phosphosites in CTL and T2D iHeps. A) PCA plot of insulin stimulated phosphorylation showing the separation of phosphoproteome data by phenotype (CTL, T2D) and sex (blue-male; red-female), phenotype (control-open; T2D-closed) and insulin treatment (insulin-stimulated- triangles; and basal- circles). B and C) Representation and quantification of exemplary phosphosites belonging to enriched pathways are examples in Class 1B, D and E) in Class 2B. Data from males and females are combined for better representation. Cells from male donors are represented by squares and female donors by circles, and color indicates individual donors. Data are presented as means ± SEM, n=8 in control; n=6 in T2D, * P<0.05,** P<0.01,***P<0.001,****P<0.0001, paired t-test analysis of intensities was performed between groups for basal vs insulin, and unpaired t-test between CTL vs T2D ± Insulin.

Supplementary Figure 4

A

Class 1C: Increased phosphorylation and emergent in T2D

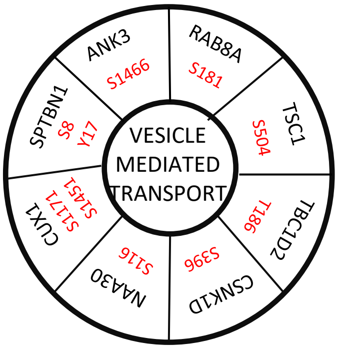

B

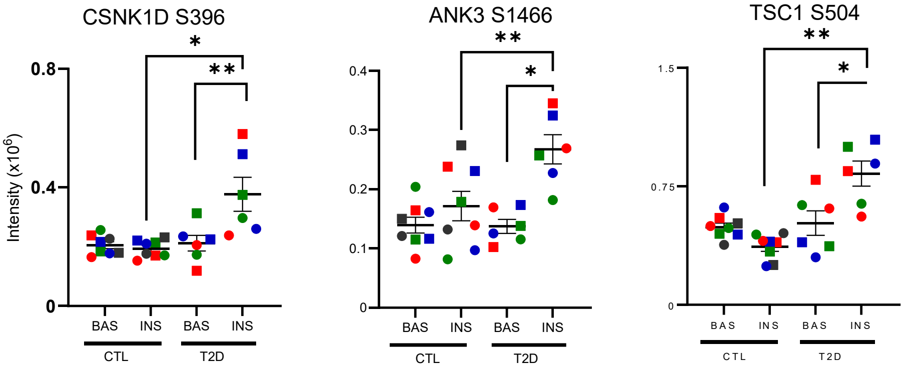

C

Class 2C: Decreased phosphorylation and emergent in T2D

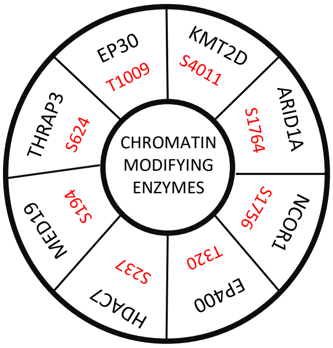

D

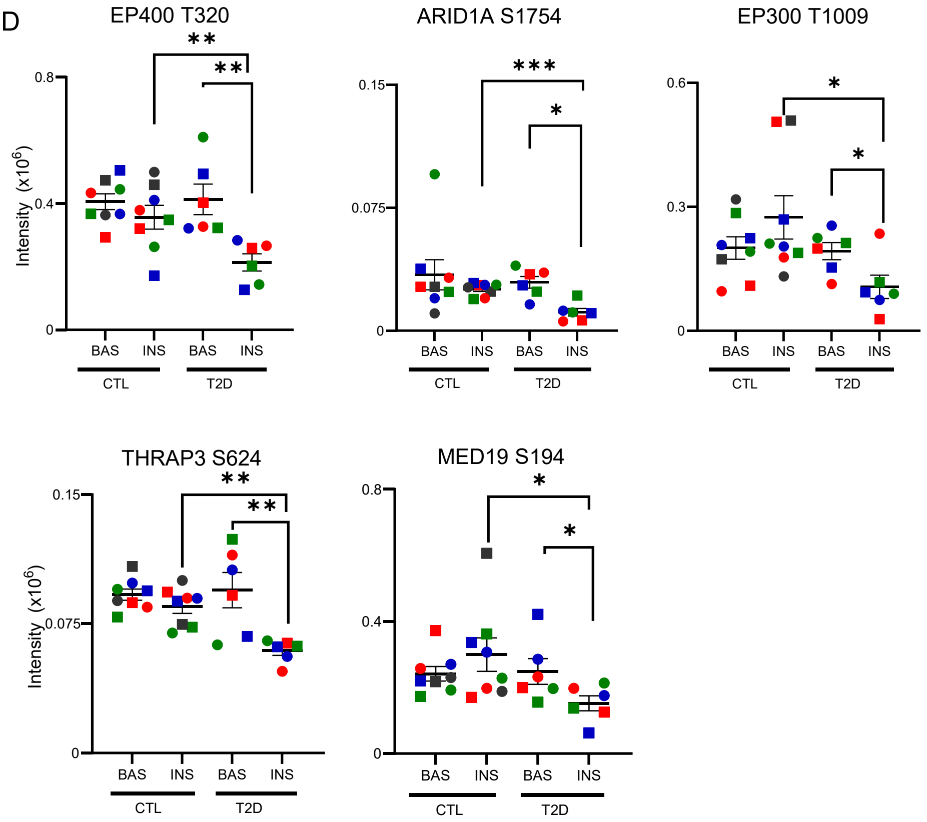

Supplementary Figure S4: Emergent phosphosites in CTL and T2D iHeps. A-B) Representation and quantification of exemplary phosphosites belonging to significantly enriched pathways are shown as examples in Class 1C, C and D) in Class 2C. Cells from male donors are represented by squares and female donors by circles, and color indicates individual donors. Data are presented as means ± SEM, n=8 in control; n=6 in T2D, * $P<0.05$,** $P<0.01$,***$P<0.001$,****$P<0.0001$, paired t-test analysis of intensities was performed between groups for basal vs insulin, and unpaired t-test between CTL vs T2D ± Insulin.

Supplementary Figure 5

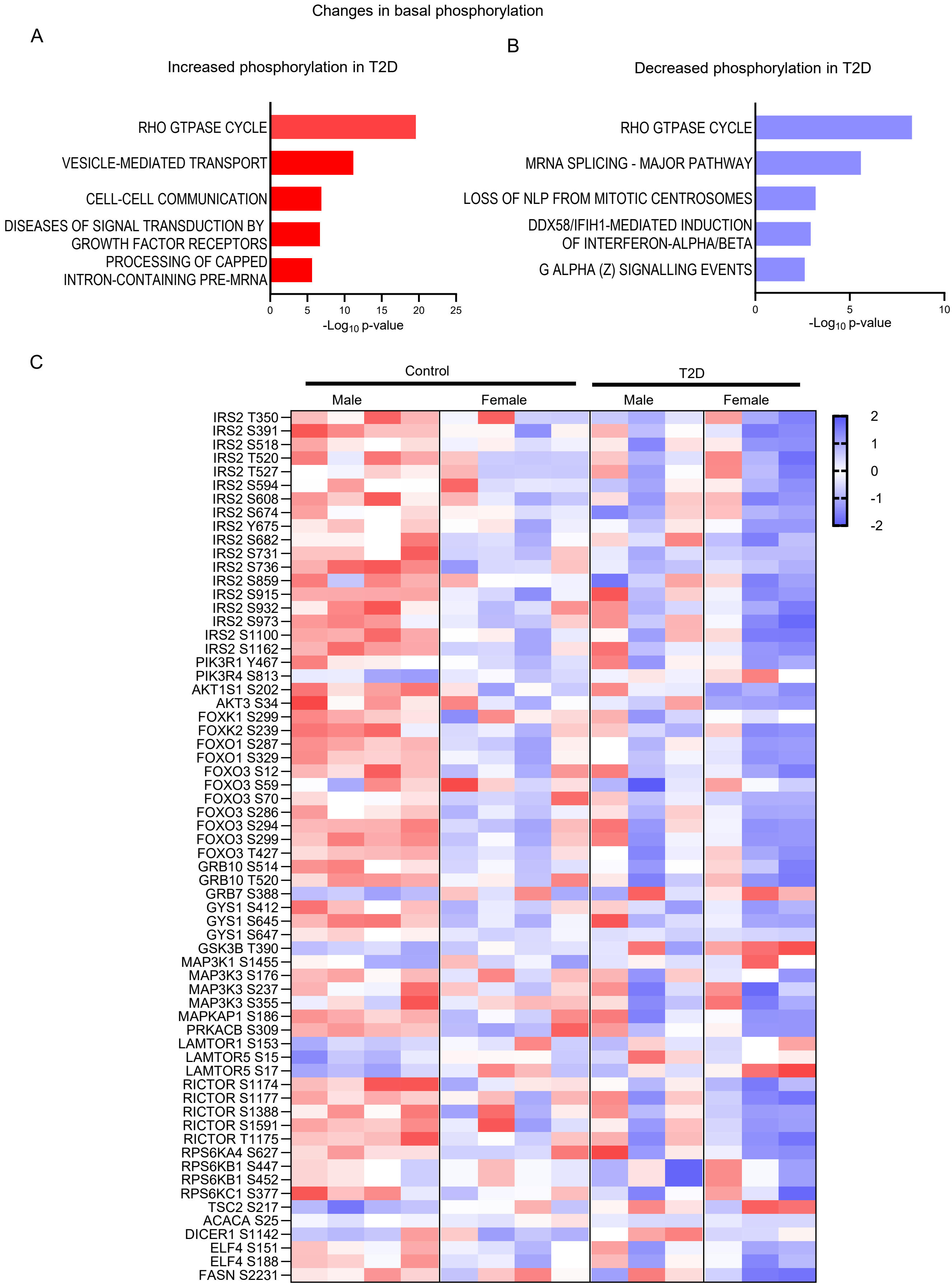

Supplementary Figure S5: Basal alterations in T2D insulin resistance. A) Reactome pathway enrichment of phosphosites that display increased phosphorylation in T2D compared to control at baseline (left) and decreased phosphorylation in T2D compared to CTL (right). B) Hierarchical heatmap analysis of significant log normalized z scores of phosphorylation comparing control iHeps and T2D iHep at the basal state (non-insulin stimulated). Data are mean, +/- SEM, unpaired t-tests between groups * $P<0.05$, ** $P<0.01$,***$P<0.001$,****$P<0.0001$.

Supplementary Figure 6

Changes in basal phosphorylation

A

RHO-GTPase cycle

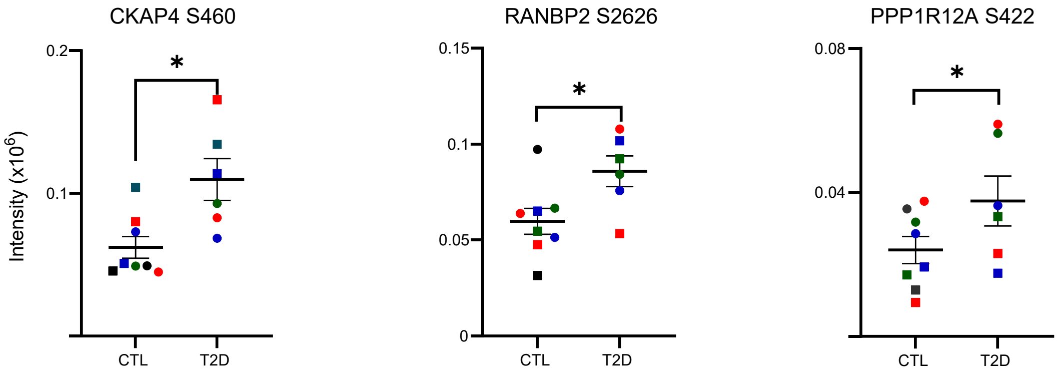

B

DDX58/IFIH1-mediated induction of interferon-alpha/beta

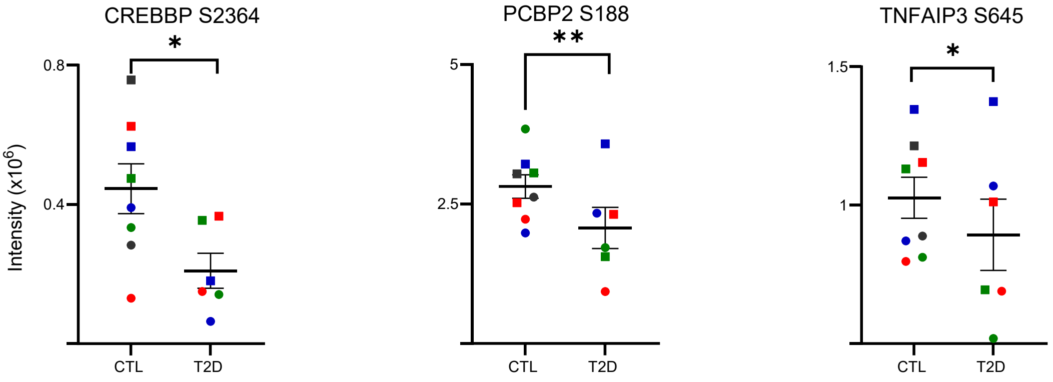

C

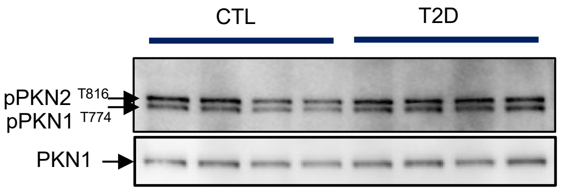

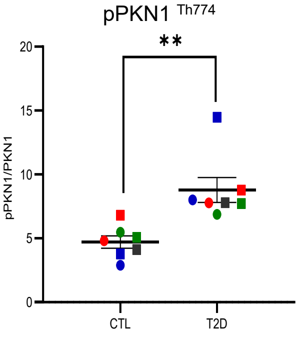

D

Sexual dimorphism in insulin signaling

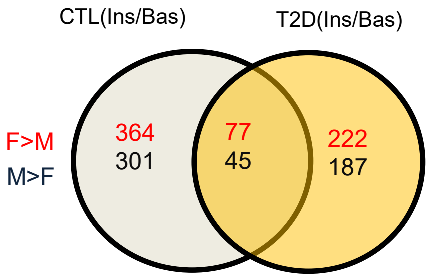

Supplementary Figure S6: Basal alterations in T2D insulin resistance and sexual dimorphism in insulin signaling in CTL and T2D. A) and B) Quantifying representative examples of increased and decreased phosphorylation in the basal state of examples from pathways that showed sexual dimorphism. Cells from male donors are represented by squares and female donors by circles, and color indicates individual donors. C) Immunoblotting of $PKN1^{T774}$ and total PKN1 in control and T2D males, and quantification of all samples on the right shown in a bar graph. Data are mean, +/- SEM, unpaired t-tests between groups * P<0.05. D) Venn diagram showing sites significantly different in insulin stimulation between sexes. Female dominant sites were defined as female>male with p <0.05; male dominant sites were defined as male>female at p-value <0.05. In both, the phosphorylation was expressed as the stimulation ratio (Ins/Bas).

Supplementary Figure 7

A

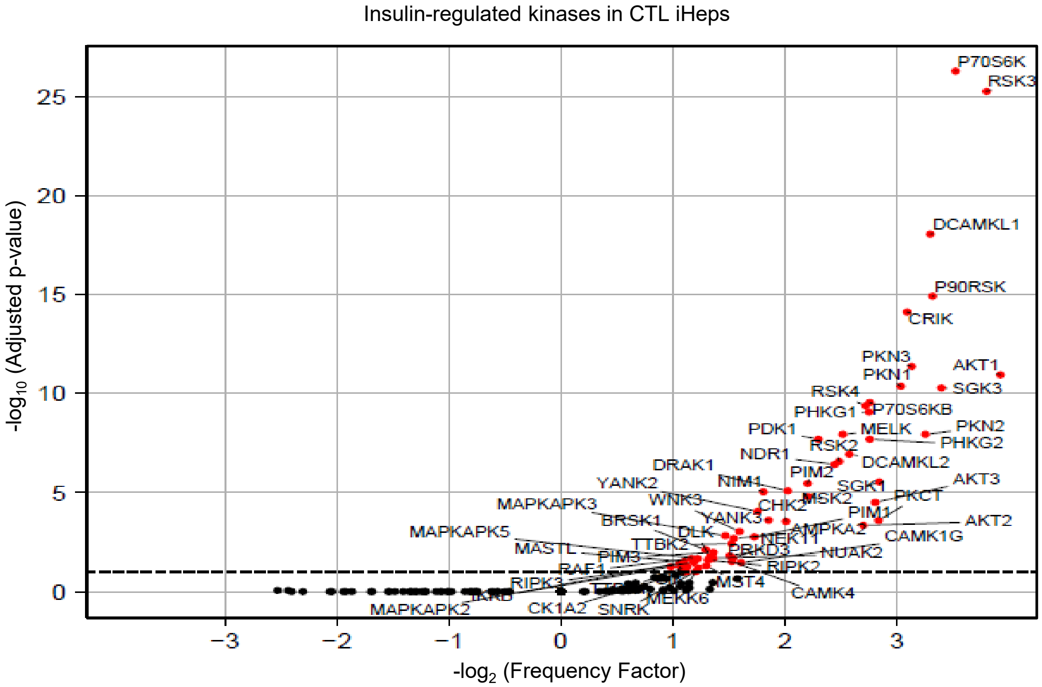

B

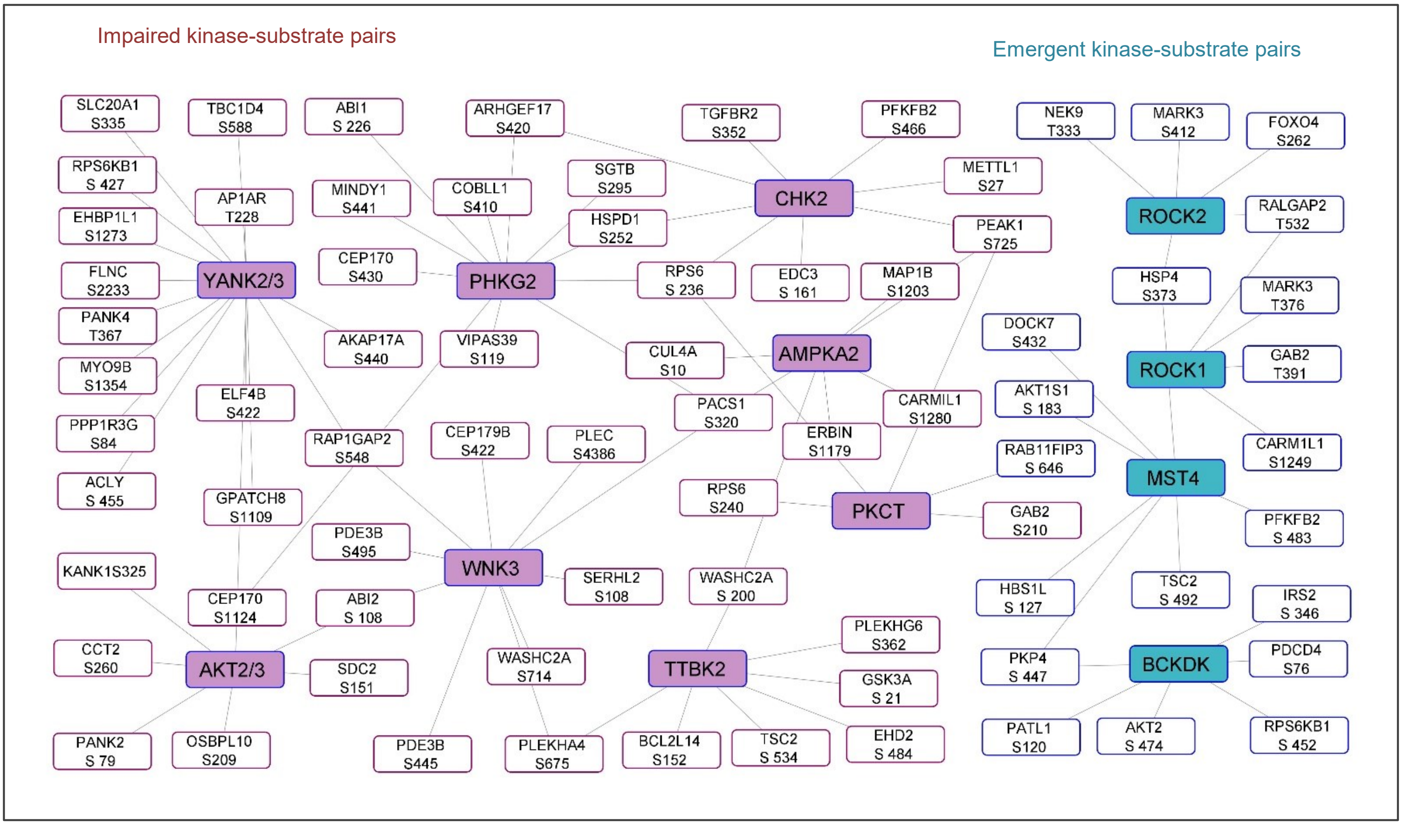

Supplementary Figure S7: Kinome profiling of predicted kinases related to insulin stimulation. A) Volcano plot showing kinases predicted to account for the altered substrate phosphorylation, increased and decreased upon insulin stimulation with plotting $\log_2$ frequency factor on the x-axis and $-\log_{10}$ p value on the y-axis. B) The top insulin-stimulated substrate-kinase pairs that showed impaired signaling in T2D are on the left side of the map (shown pink), and the kinases and phosphosites of emergent signaling in T2D on the right side of the map (blue).

Supplementary Figure 8

A

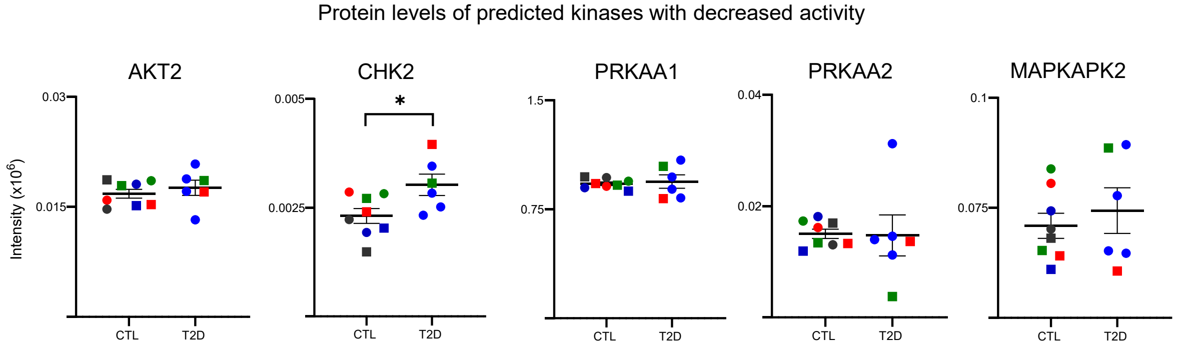

B

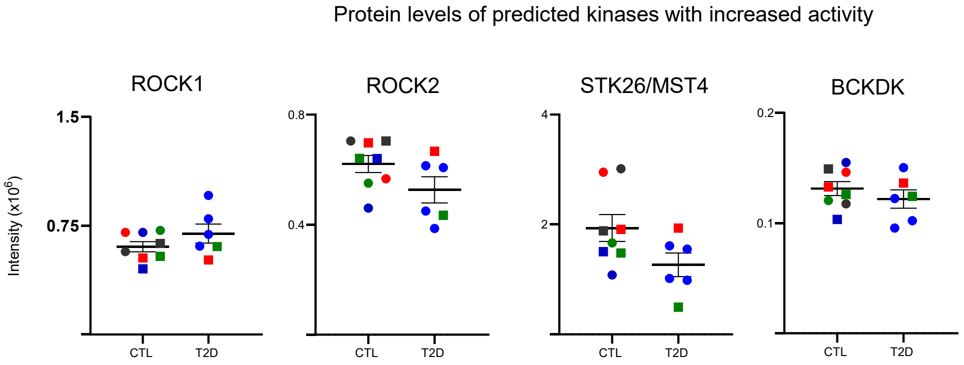

Supplementary Figure S8: Defining kinase insulin regulated substrate phosphorylation relationship in T2D iHeps. A) Protein levels of representative kinases detected in the LC-MS/MS proteomics predicted to be involved in impaired and emergent signaling in T2D. Cells from male donors are represented by squares and female donors by circles, and color indicates individual donors. Data are mean, +/- SEM, unpaired t-tests between groups * $P<0.05$, ** $P<0.01$,*** $P<0.001$,**** $P<0.0001$.

Supplementary Figure 9

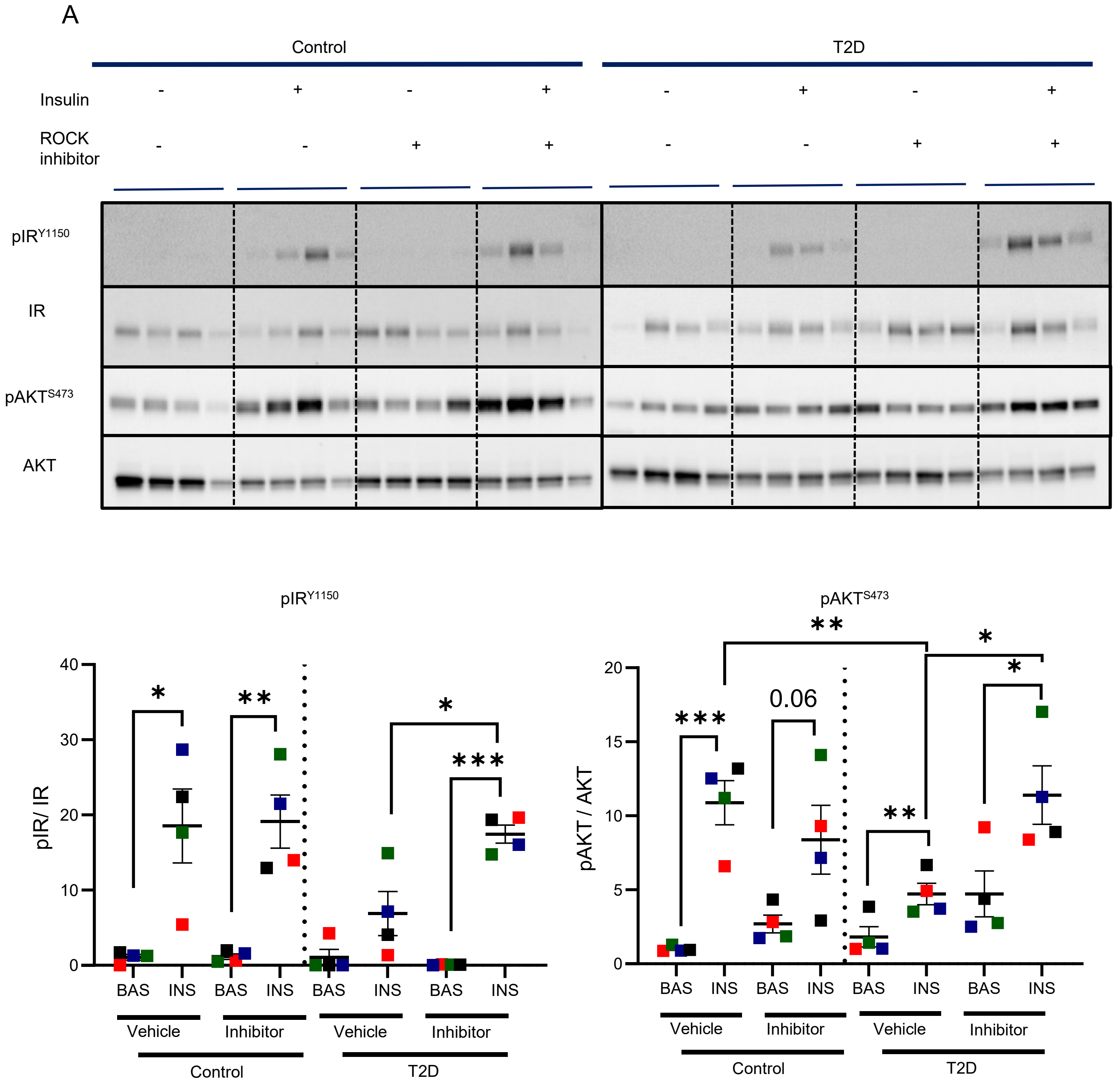

Supplementary Figure S9: Inhibition of ROCK in control and T2D iHeps. B) Representative immunoblot analysis of phosphorylation of proteins in four control and four T2D male donors' with and without 100 nM insulin and 10 nM Ripasudil. Quantification of the data in bar graphs normalized to a total protein is on the right. Cells from male donors are represented by squares and color indicates individual donors Data means +/- SEM, n=6-8, * $P<0.05$, ** $P<0.01$, Basal vs Insulin, Insulin vs Insulin+inhibitor, paired t-test and unpaired t-test for CTL Insulin vs CTL Inhibitor, T2D Insulin vs T2D Inhibitor, CTL Insulin vs T2D Insulin. The immunoblotting and quantification data shown in Figure 6B is from the T2D iHeps, while complete data from control and T2D are shown again here.

Supplementary Methods:

FACS analysis:

iHeps from control and T2D were grown on 12-well plates and differentiated as described above for immunostaining. The cells were permeabilized with 0.1% Triton-X and blocked with 10% normal goat serum for 10 minutes. Cells were washed with 0.01% Triton-X 100 containing PBS twice and incubated with 1:1500 anti-albumin antibody (Millipore) dilution for one hour at RT. Cells were also stained with propidium iodide (PI) for viability. The cells were stained with appropriate secondary antibodies for one hour and washed with PBS before FACS analysis.

Albumin Immunostaining:

iHeps from control and T2D donors were grown on 6 well plates and differentiated as described above. Tor immunostaining, the cells were permeabilized with 0.1% Triton-X and blocked with 10% normal goat serum for 10 minutes, then incubated with 1:500 anti-albumin antibody (Millipore) dilution for one hour at RT. Cells were also stained with DAPI for nuclear staining.
