# Supplementary figures and images for: Cell-intrinsic insulin signaling defects in human iPS cell–derived hepatocytes in type 2 diabetes
Source: J Clin Invest. 2025 Apr 15;135(8):e183513. doi: 10.1172/JCI183513 (PMC11996863; doi:10.1172/JCI183513)

Full blots/gels from Figure 1E

D

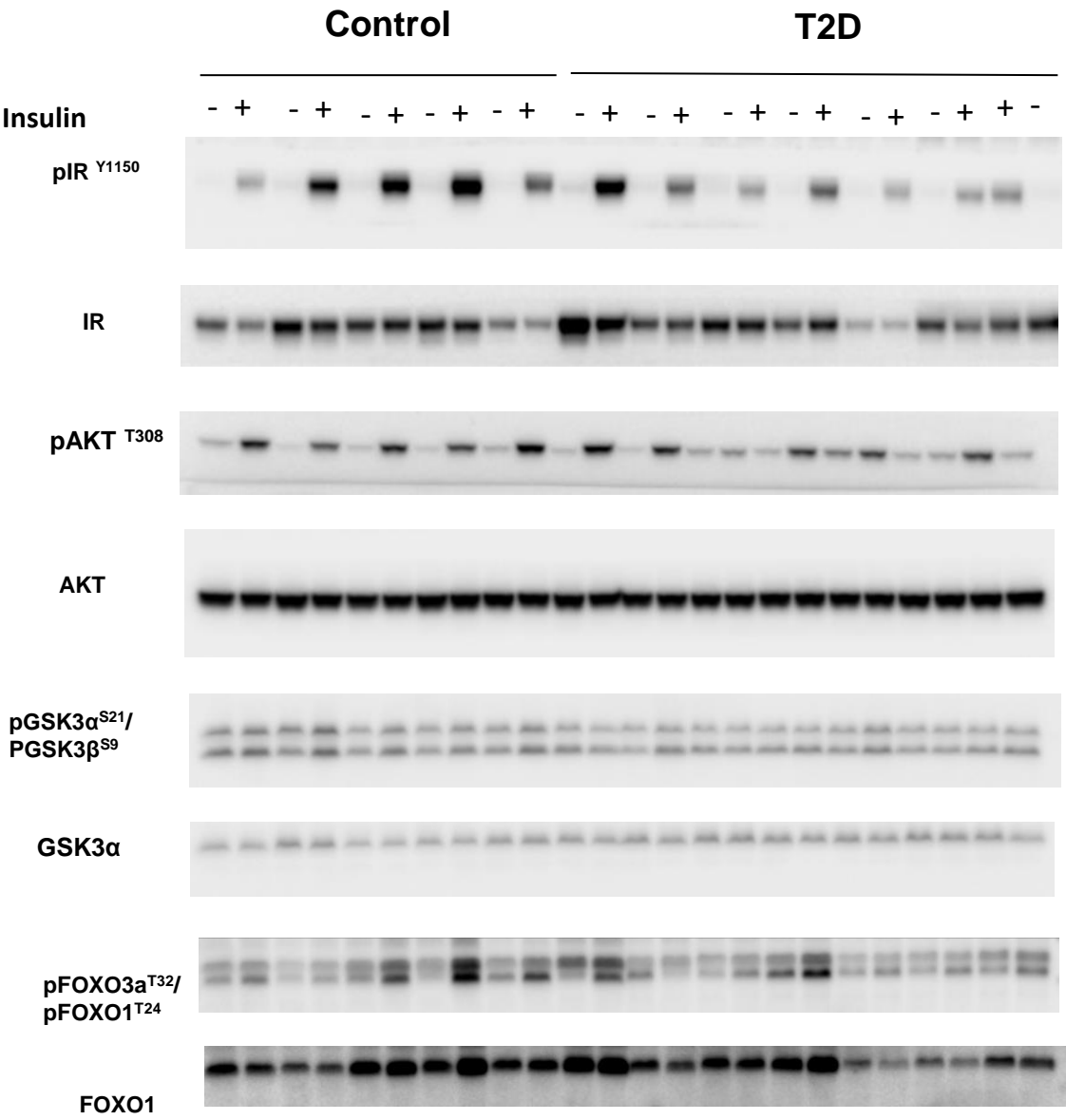

Full blots/gels from Figure 6B

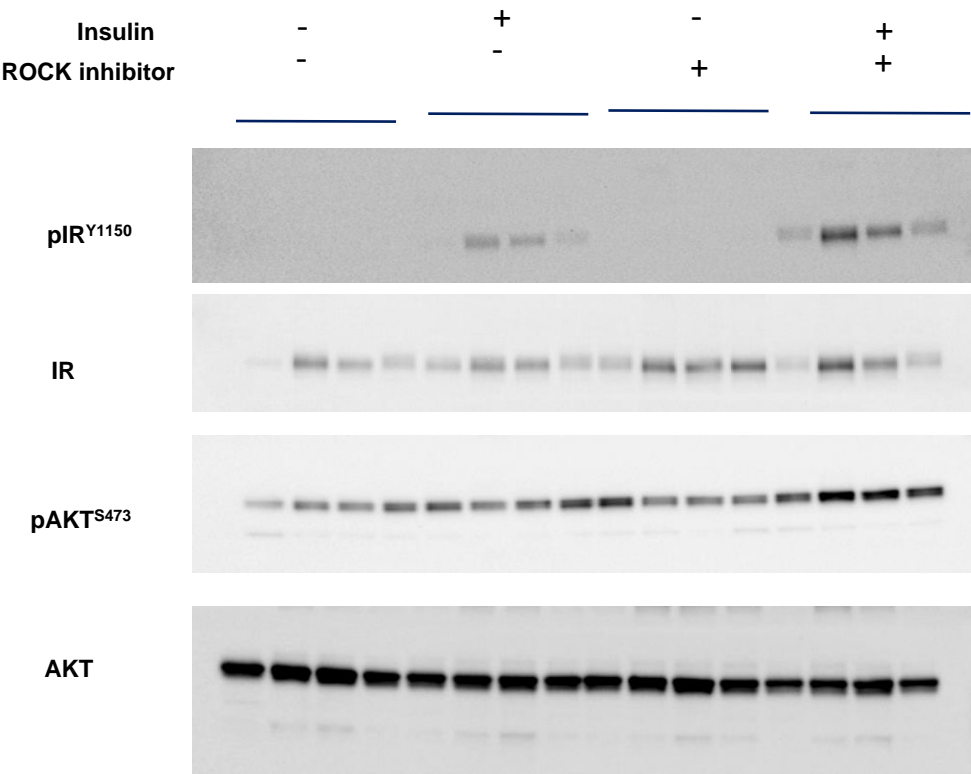

Supplement: Unedited blot and gel images [file jci-135-183513-s175.pdf]
